# Groundwater Throughflow and Seawater Intrusion in High Quality Coastal Aquifers

**DOI:** 10.1038/s41598-020-66516-6

**Published:** 2020-06-17

**Authors:** A. R. Costall, B. D. Harris, B. Teo, R. Schaa, F. M. Wagner, J. P. Pigois

**Affiliations:** 1 Western Australian School of Mines: Minerals, Energy and Chemical Engineering, Curtin University, Curtin, Western Australia; 2 Institute for Applied Geophysics and Geothermal Energy, RWTH Aachen University, Aachen, Germany; 3 Department of Water and Environmental Regulation (DWER), Joondalup, Western Australia

**Keywords:** Environmental impact, Hydrology, Natural hazards, Fluid dynamics

## Abstract

High quality coastal aquifer systems provide vast quantities of potable groundwater for millions of people worldwide. Managing this setting has economic and environmental consequences. Specific knowledge of the dynamic relationship between fresh terrestrial groundwater discharging to the ocean and seawater intrusion is necessary. We present multi- disciplinary research that assesses the relationships between groundwater throughflow and seawater intrusion. This combines numerical simulation, geophysics, and analysis of more than 30 years of data from a seawater intrusion monitoring site. The monitoring wells are set in a shallow karstic aquifer system located along the southwest coast of Western Australia, where hundreds of gigalitres of fresh groundwater flow into the ocean annually. There is clear evidence for seawater intrusion along this coastal margin. We demonstrate how hydraulic anisotropy will impact on the landward extent of seawater for a given groundwater throughflow. Our examples show how the distance between the ocean and the seawater interface toe can shrink by over 100% after increasing the rotation angle of hydraulic conductivity anisotropy when compared to a homogeneous aquifer. We observe extreme variability in the properties of the shallow aquifer from ground penetrating radar, hand samples, and hydraulic parameters estimated from field measurements. This motived us to complete numerical experiments with sets of spatially correlated random hydraulic conductivity fields, representative of karstic aquifers. The hydraulic conductivity proximal to the zone of submarine groundwater discharge is shown to be significant in determining the overall geometry and landward extent of the seawater interface. Electrical resistivity imaging (ERI) data was acquired and assessed for its ability to recover the seawater interface. Imaging outcomes from field ERI data are compared with simulated ERI outcomes derived from transport modelling with a range of hydraulic conductivity distributions. This process allows for interpretation of the approximate geometry of the seawater interface, however recovery of an accurate resistivity distribution across the wedge and mixing zone remains challenging. We reveal extremes in groundwater velocity, particularly where fresh terrestrial groundwater discharges to the ocean, and across the seawater recirculation cell. An overarching conclusion is that conventional seawater intrusion monitoring wells may not be suitable to constrain numerical simulation of the seawater intrusion. Based on these lessons, we present future options for groundwater monitoring that are specifically designed to quantify the distribution of; (i) high vertical and horizontal pressure gradients, (ii) sharp variations in subsurface flow velocity, (iii) extremes in hydraulic properties, and (iv) rapid changes in groundwater chemistry. These extremes in parameter distribution are common in karstic aquifer systems at the transition from land to ocean. Our research provides new insights into the behaviour of groundwater in dynamic, densely populated, and ecologically sensitive coastal environments found worldwide.

## Introduction

Mankind has always interacted with the natural environments at coastal margins. Beaches, sand dunes, limestone cliffs, and the ocean are part of daily life for vast numbers of people. Hidden beneath the surface exists the interplay between a dense wedge of saline groundwater fuelled from the sea, and fresh terrestrial groundwater driving towards the ocean. We explore this relationship with a focus on methods for quantifying the relationship between terrestrial groundwater flowing toward the ocean and the landward extent of the seawater wedge for high-quality aquifers.

Any reduction in fresh groundwater flowing towards the ocean can potentially cause seawater intrusion. This impacts private bores, irrigation systems, and access to potable water^1–3^. It can also affect sensitive near-shore ecosystems that rely on the nutrients supplied from terrestrial submarine groundwater discharge^4–7^. This can alter groundwater chemistry with significant environmental and economic consequences^8–12^. Research into monitoring coastal groundwater systems and the seawater wedge provides inputs to managing and maintaining healthy coastal aquifers and ecosystems.

Current monitoring practices rely on wells for information on groundwater levels, chemistry, provenance, and age. These data are needed to build numerical groundwater models suitable to predict the consequences of water resource decisions. Drilling and wireline logging are typically used to infer lithology and hydraulic properties. However, this information tends to be localised and can have a dependence on the design of the well or wellfield^13^.

This research systematically traverses the challenges and opportunities faced in monitoring the seawater interface in a complex coastal setting. It combines elements from hydrogeology, well-based monitoring technologies, analytical seawater interface solutions, solute transport modelling of increasing complexity, and geophysical methods.

**Figure 1:**
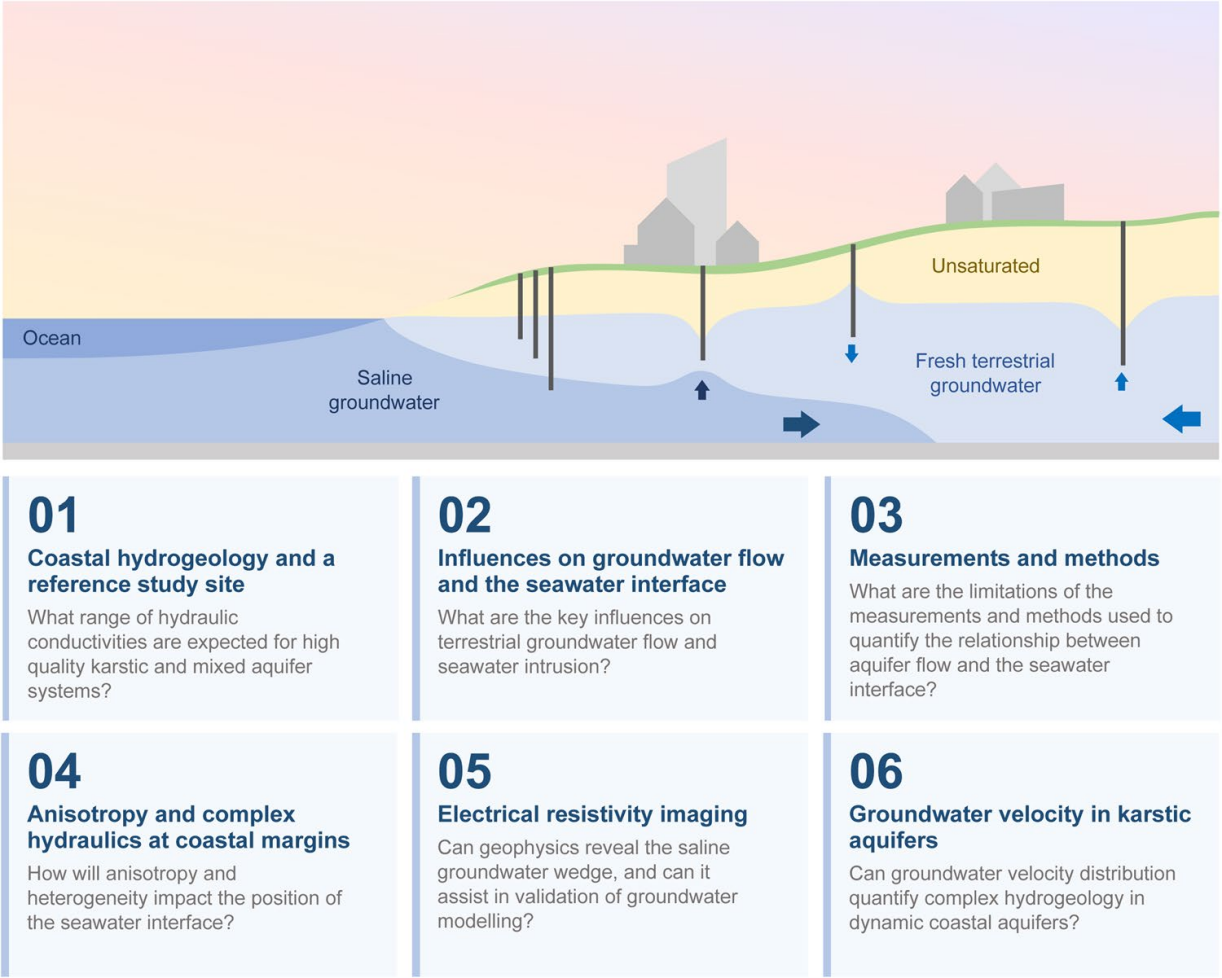
Schematic of a shallow coastal groundwater water system in an urban setting and our research outline. The multidisciplinary research is partitioned into six connected parts spanning geology, method of acquisition and processing of well data, numerical groundwater modelling and geophysical methods. An example of the type of questions addressed in each part is provided.

The overview shown in Fig. 1 assists the reader in navigating our research, and includes a schematic showing the geometric relationship between groundwater wells and the seawater interface in an urban setting. An example of the type of questions addressed within each part is also provided in Fig. 1. Each part of our research is summarized below.

- Part 1: Here we introduce the elements of a coastal karstic aquifer system and our seawater intrusion reference site before comparing it to the hydrogeology of karstic aquifers found worldwide. We include examples of limestone sourced from the reference site and illustrate heterogeneity and dip of the shallow geology using ground penetrating radar.
- Part 2: In this part we identify the influences that may impact the seawater interface at the reference site. The site has experienced significant changes in the position of the seawater interface and groundwater hydraulics throughout the monitoring period. This important site motivates our research concerning methods for characterising groundwater throughflow and the seawater interface along shallow coastal aquifer systems.
- Part 3: Part 3 presents the data from the reference site and systematically explores the potential for error in predicting seawater intrusion from groundwater flow using conventional monitoring techniques. Examples from our reference site lead us more sophisticated numerical modelling.
- Part 4: Here we simulate the impacts of anisotropy and heterogeneity on a coastal aquifer. We investigate the impact of dipping hydraulic conductivity anisotropy on the relationship between the landward position of the seawater interface toe and the rate of groundwater flow towards the ocean. Our numerical experiments extend to complex, extremely heterogeneous hydrogeology as found at our seawater intrusion reference site.
- Part 5: This part explores the ability of geophysical techniques to accurately image the seawater interface and coastal hydrogeology. Electrical resistivity imaging (ERI) has the potential to estimate the distribution of salinity in time and space. However, imaging outcomes from ERI can be over-interpreted and we consider the implementation and practicality of ERI for recovering the seawater interface for karstic systems and the reference site.
- Part 6: In Part 6 we focus on the dramatic changes in groundwater velocity that occur proximal to the seawater interface. These details have implications for both short-term and long-term monitoring strategies and solutions. This leads us toward options for new integrated monitoring systems that are tailored to shallow high-quality coastal aquifers.

## Part 1. Coastal Hydrogeology and a Reference Site

### Introduction to the near-shore coastal margin

**Figure 2:**
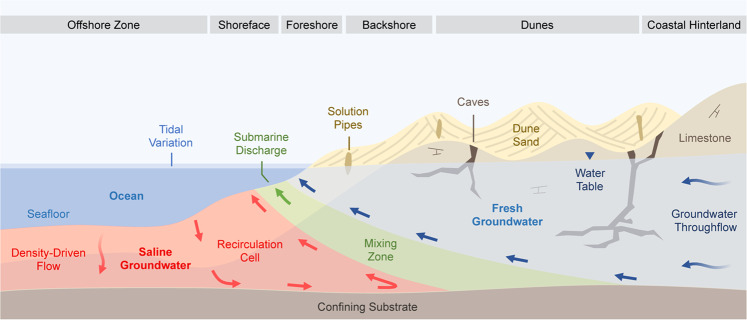
Schematic of coastal hydrogeology indicating processes and nomenclature for the seawater interface in a mixed siliciclastic and carbonate near-shore aquifer system. The permeability distribution, groundwater throughflow, and density-driven flow combine to determine the geometry of the seawater/freshwater mixing zone, patterns of groundwater flow within the seawater recirculation cell, and the distribution of submarine discharge. Temporal cycles, such as the seasonal rainfall, pumping from shallow wells, and tidal forcing drive constant groundwater movement in the coastal aquifers. ‘Groundwater throughflow’ describes the volume of groundwater entering the system and flowing towards the coast.

The near-shore coastal margin has nomenclature to describe the specific depositional environments and groundwater processes^14^. A schematic of this terminology is shown in Fig. 2, and includes features that are commonly associated with recent (Pleistocene) karstic aquifers such as solution pipes and cave systems^15,16^. The geometric arrangement and distribution of these post-depositional features may play a significant role in determining the geometry of the seawater interface.

At the coastal margin, higher density seawater (~1025 kg/m³) sinks beneath the less dense terrestrial groundwater (~1000 kg/m³) forming a wedge shape^17,18^. The mixing zone describes the area where these two waters interact and form a solute gradient^19^. The extent of this zone can be highly variable, ranging from sub-meter scales to multiple kilometres^20–22^, and is chemically-active, with implications for limestone dissolution and re-cementation^23,24^.

Groundwater throughflow and hydraulic conductivity are often used to predict the inland position of the seawater wedge, referred to as the ‘toe’. Groundwater throughflow describes the volume of water flowing in the terrestrial aquifer system towards the coast^25^. For our cross-sectional model, volume calculations are based on a boundary with unit length (1 m), such that groundwater throughflow is expressed in units of ML/year/m (e.g. see Part 3).

In addition to the zones shown in Fig. 2, there are many temporal changes that influence the position of the seawater interface. These include effects of wave surges, beach geometry, tides, rainfall recharge, and groundwater abstraction. These variables ensure that solute distribution and hydraulic heads along coastal margins are in constant motion. In high-quality karstic aquifers, these temporal challenges are compounded by the variability in hydraulic properties associated with high-permeability caves, low-permeability cemented limestone, and anisotropy of hydraulic conductivity.

Supplementary Table S1 contains a summary table of spatial and temporal parameters that can affect the position and geometry of the seawater wedge. We will provide specific examples illustrating the potential impact of heterogeneity and anisotropy of hydraulic conductivity on the seawater wedge in Part 4.

### An urban reference site where seawater intrusion has occurred

Increase in global population density at coastal margins and associated demand for high-quality, low-cost water can significantly impact coastal aquifer systems. Our research site in Quinns Rocks, approximately 35 km north of Perth, Western Australia, has experienced a rapid increase in population and urban development during the period of monitoring. Perth, the capital of Western Australia, is one of the lowest population density cities worldwide (~323 persons per square kilometre^26,27^). However the majority of urban sprawl is occurring along the coastal margins^28^.

**Figure 3:**
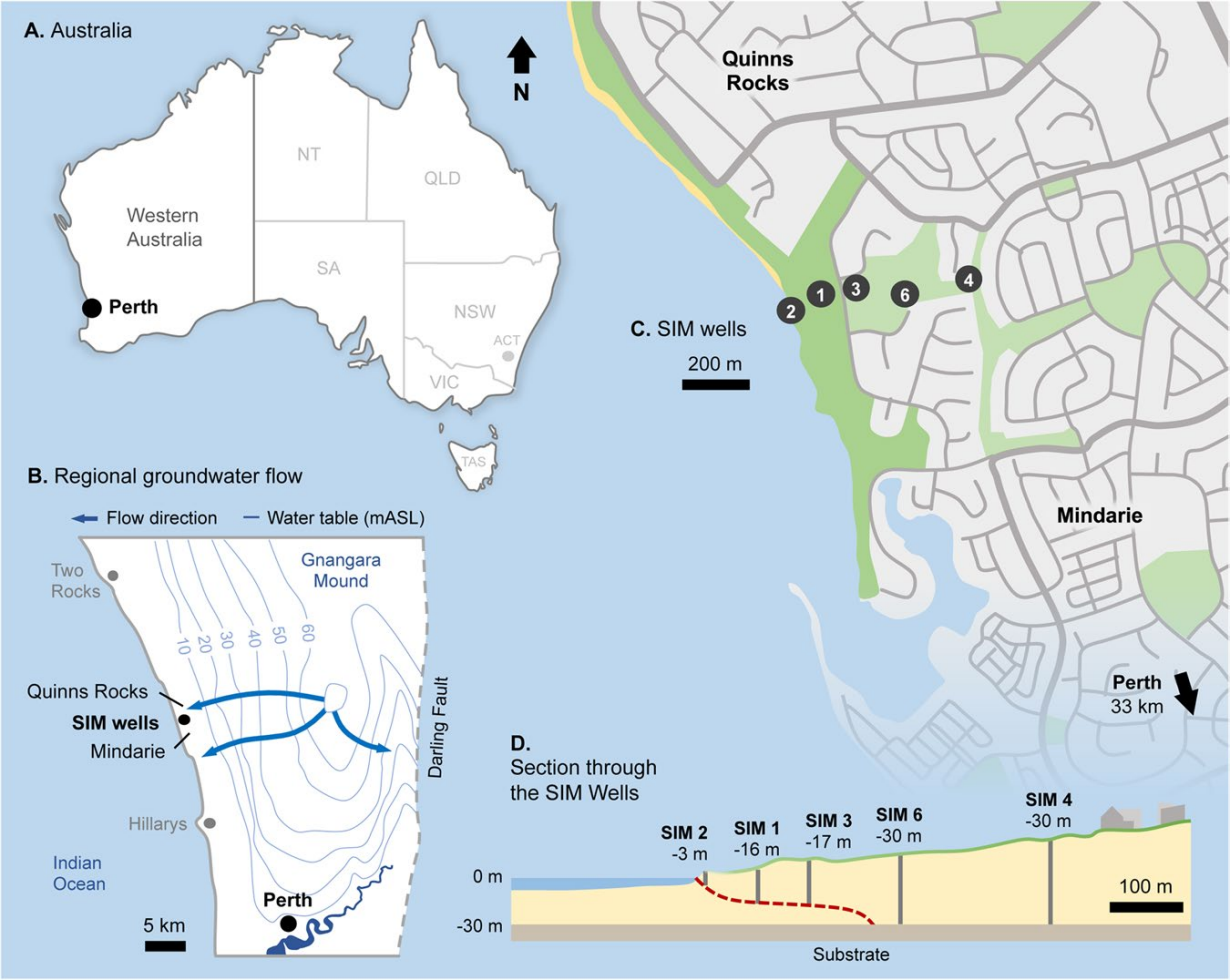
Maps providing location and characterisation of the Quinns Rocks seawater intrusion monitoring (SIM) reference site in Perth, Western Australia. (**A**) The location of Perth relative to Western Australia. (**B**) The regional groundwater contours, which are approximately parallel to the coastline at Quinns Rocks indicating groundwater flows towards the coast. (**C**) The location of the SIM wells relative to Quinns Rocks, which have been monitored since 1990. (**D**) A cross-section of the SIM wells, including the impermeable clayey substrate and an estimate of the current position of the seawater wedge.

The location of the seawater intrusion monitoring (SIM) wells at Quinns Rocks is shown in Fig. 3. It includes the minimum regional groundwater level contours in the upper superficial aquifer for May 2003^29^. It also includes a cross-section through the SIM wells and the approximate position of the seawater interface in 2018. Regional groundwater flow is perpendicular towards the shoreline^30^. The shallow hydraulic gradients of the regional water level contours approaching the shoreline suggest high permeability in the mixed limestone aquifers typical of Perth’s coastal margin^31,32^. The details of the SIM well completions are found in Table 1, including the location and depth of the screened intervals.

**Table 1:** Details of the SIM wells including approximate distance from shoreline and depth of the screens below ground level (m BGL).

Well	Easting	Northing	Distance from	Ground Level	Screen Depth (m BGL)		Mean Screen Depth
ID	mE	mN	Shoreline (m)	(m AHD)	From	To	(m AHD)
SIM 2	376567.43	6494093.94	30	6.13	7.85	8.85	−2.22
SIM 1	376635.39	6494122.71	105	11.04	26.1	27.1	−15.56
SIM 3	376719.26	6494142.05	190	15.18	31.87	32.87	−17.19
SIM 6	376900.26	6494133.29	360	24.03	53.58	54.58	−30.05
SIM 4	377084.02	6494174.60	550	31.34	60.43	61.43	−29.59

### The shallow geology and hydraulics at coastal margins

A fundamental step in creating a valid and practical groundwater model is to analyse local hydrogeology. Groundwater management often relies on predictive groundwater modelling to determine the future impact of groundwater allocations and water supply options. In karstic groundwater systems, aquifer hydraulics can be highly variable over short distances^33–35^. Localised high-permeability conduits coupled with extremely low-permeability layers of cemented limestone can form strongly anisotropic aquifer systems^34,36^. These environments can influence the shape of the seawater interface and be associated with unconventional seawater wedge geometries^21,37–39^.

**Figure 4:**
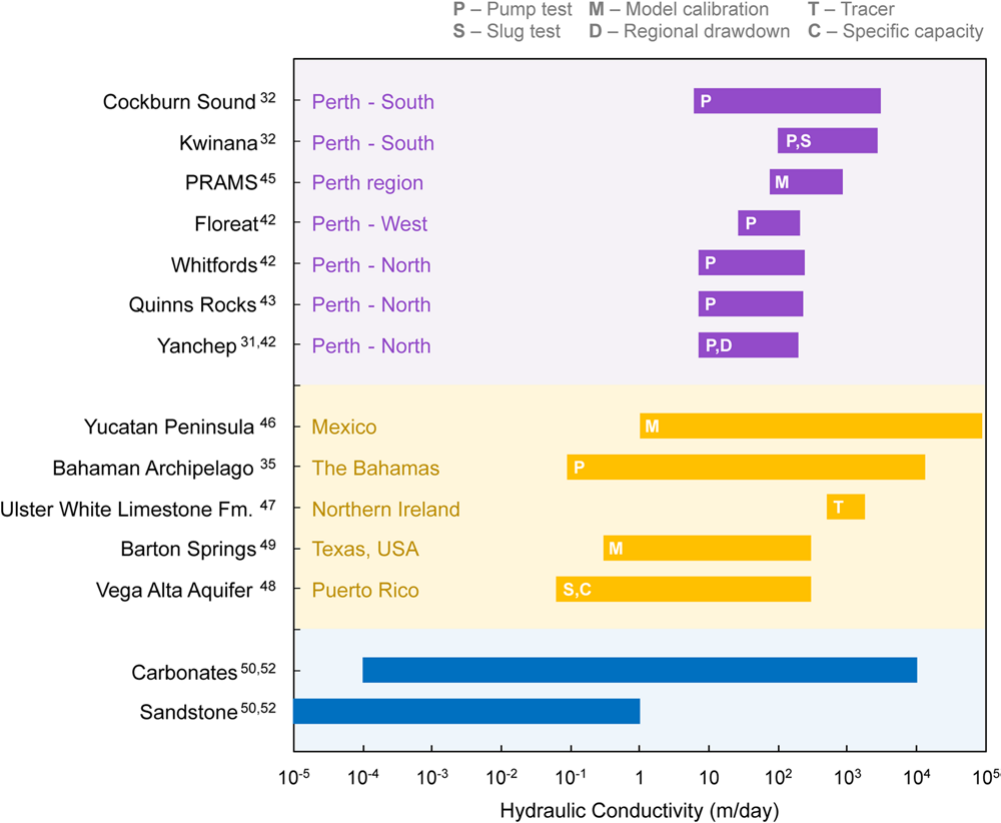
Ranges of hydraulic conductivity estimated at sites along the coastal margin of Perth (shown in purple) and other karstic environments around the world (shown in yellow). Most of the hydraulic conductivity in the Perth region fall within the mid-to-upper estimate of typical carbonate values (shown in blue). The karstic aquifers in Perth is known to contain caves and other high-permeability flow pathways that contribute to the high hydraulic conductivity.

The range of estimated values for hydraulic conductivity found in literature examples from around the world are compared with examples from the coastal margin of Perth in Fig. 4^22,32,35,40–50^. The hydraulic conductivity of karstic aquifers can be orders of magnitude higher than clastic aquifers^51,52^. The hydraulic conductivity along Perth’s coastal margin is estimated to be between 10 to 10000 m/day, and the estimated average at the Quinns Rocks reference site is between 130–200 m/day^53^. Further detail on the estimates local to Quinns Rocks can be found in Supplementary Tables S2, S3 and S4.

In Perth, the shallow aquifer is dominantly comprised of the Pleistocene-Holocene-aged Tamala Limestone. The Tamala Limestone is characterised by shallow horizontal cave systems, a lack of directed conduits, cave clustering near the coast, and extensive collapse-dominated cave systems^16,54,55^. Evidence for these systems is found in outcrops, accessible cave systems, limestone quarries, and high-resolution ground penetrating radar. These features introduce a range of challenges in hydraulic modelling, particularly for numerical groundwater model calibration^31,42,56^.

**Figure 5:**
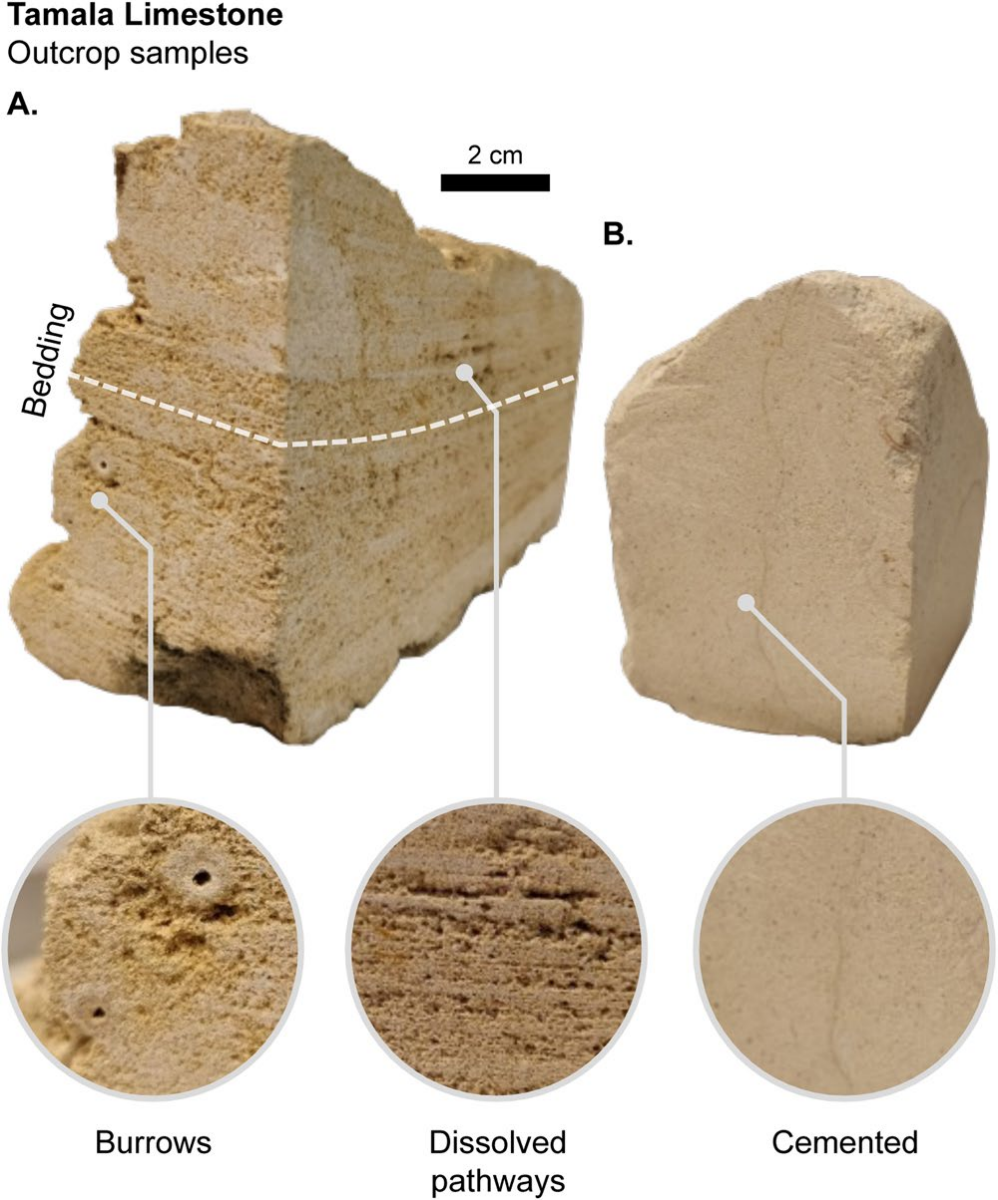
Photographs showing samples of the Tamala Limestone with different sedimentary fabrics. (**A**) A vuggy sample with burrows and dissolved pathways, expected to be highly permeable. (**B**) A cemented massive fine-grained limestone sample, expected to have considerably lower permeability. This figure highlights the contrast in geological fabrics that directly impact hydraulic conductivity. Large-scale karsts (i.e. caves and other conduits) exist throughout the coastal margin of Western Australia.

Photographic examples of geological fabrics common in the Tamala Limestone are shown in Fig. 5. The sample labelled Fig. 5B is typical of a well-cemented limestone, and the sample labelled Fig. 5A is a typical of a highly porous, high-permeability example of Tamala Limestone. This permeable sample (Fig. 5A) shows preferential dissolution along bedding, which can form extremely high hydraulic conductivity networks throughout some layers of the aquifer. In contrast, the sample shown in Fig. 5B is fine grained, well cemented, and likely to have significantly lower hydraulic conductivity.

**Figure 6:**
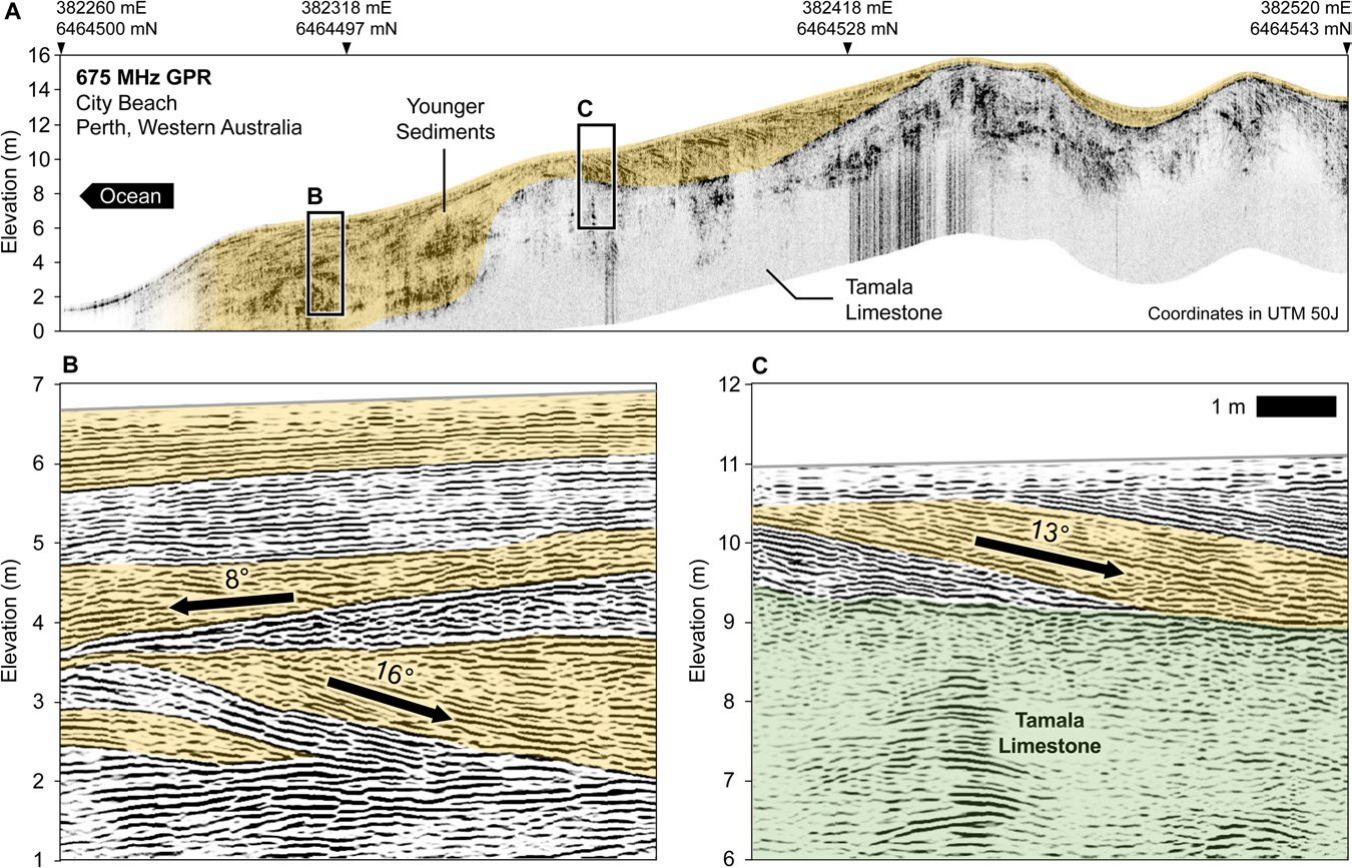
A ground penetrating radar (GPR) section at Perth’s coastal margins that expresses the typical beach and dune facies and limestone ridges. Panel A shows the GPR energy envelope attribute that highlights the unconformity between the Tamala Limestone and beach/dune facies above. Panel B is a GPR section showing the reflections from sedimentary layering present in the younger facies, with a prevailing dip towards the ocean. Panel C is a GPR section showing the reflections from sedimentary layers dipping approximately 13° landwards (away from the coast) and terminating at an unconformity. This data was collected at the suburb of City Beach, south of Quinns Rocks, using a 675 MHz antenna. Similar examples of dipping beds and limestone ridges occur throughout the coastal margin of Perth^15,57,58,187^.

Geological facies at the coastal margin can vary within tens of metres of the coastline. For example, Fig. 6 presents a high-resolution 675 MHz ground penetrating radar (GPR) profile from City Beach, 10 km west of Perth CBD. Similar images have been obtained at many locations along Perth’s coastal margin^57–60^. The GPR data reveals shallow dipping layers of modern beach and dune facies nested between limestone ridges. The dip of layers in the beach facies are estimated to be up to 16 degrees. Figure 6A shows an example of layers dipping towards the ocean (i.e. ~8° west), while Fig. 6B shows layers oriented away from the ocean (dips between 13–16° east). Processing steps and parameters for the GPR data can be found in Supplementary Table S5.

Two important conclusions stem from the analysis of the karstic near shore setting of the Perth region. These are: (i) extreme changes in lithological character and associated hydraulic parameters are common, especially perpendicular to the shoreline, and (ii) geological layering is common and likely associated with anisotropy of hydraulic conductivity throughout Perth’s coastal margin. The influences of anisotropy and complex hydraulic conductivity distributions are analysed using numerical solute transport modelling later in this research (i.e. Part 4).

## Part 2. What drives the relationship between groundwater throughflow and Seawater Intrusion?

Three influences commonly linked with seawater intrusion include greater net groundwater use (e.g. from abstraction wells), changes in net vertical flux entering the groundwater system (e.g. rainfall recharge, storm water infiltrations etc.), and sea-level rise^20,61–65^. Data was acquired at the Quinns Rocks reference site during a 30-year period, where all three of these factors may have contributed to fundamental changes in the geometry of the seawater interface.

We consider each of these factors with reference to available data for the Quinns Rocks reference site. Our suspicion is that, although the dataset is extensive and spans several decades and wells, isolating any one of these factors is not possible with the data that was collected. Decoupling the relative contributions of each will likely require new monitoring practices.

### Rapid urbanisation and increased groundwater abstraction at the coastal margins

Groundwater abstraction leading to reduction in terrestrial groundwater flows is often identified as the primary driver for seawater intrusion^66–73^. Sustainable groundwater management initiatives, such as augmenting rainfall recharge from urban surfaces, and managed aquifer recharge are being developed to reduce the direct dependence on shallow groundwater^74–80^. Systems of aquifer replenishment such as these, introduce another dimension to groundwater flow and reinforce the need for better groundwater monitoring solutions that are better able to decouple multiple influences.

The suburbs surrounding the Quinns Rocks reference site have undergone rapid urbanisation and population growth since 1984. The SIM wells were established to assess the impact of development and expansion of Perth’s regional Integrated Water Supply Scheme (IWSS). In total, the IWSS supplies ~289 GL/year to Perth and remote mining towns^81^. This water is sourced from approximately 43% groundwater, 39% desalinated water, and 18% from surface water^82^.

**Figure 7:**
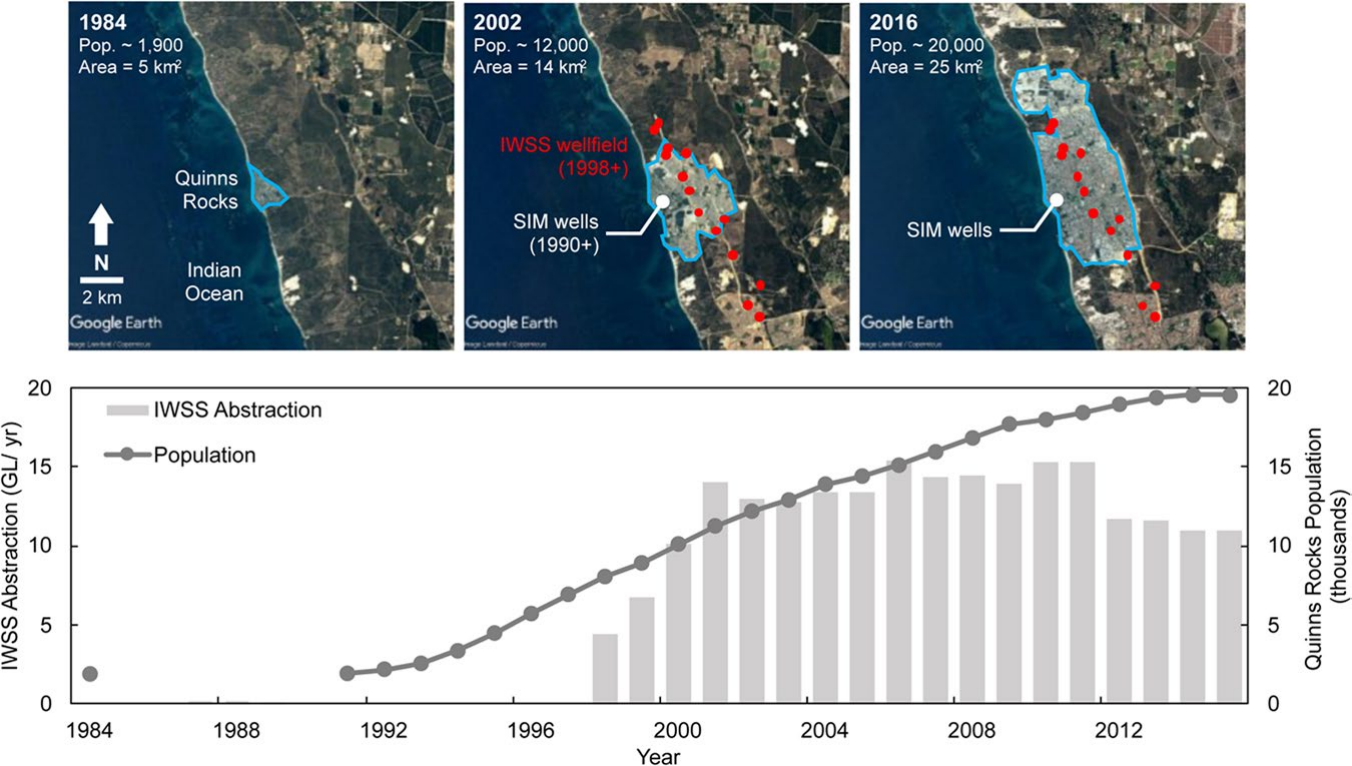
Set of satellite images showing the rapid expansion of the Quinns Rocks suburb, taken in 1984 (top left), 2002 (top middle) and 2016 (top right), and groundwater abstraction from the IWSS bore field from 1984 to 2016 (bottom). The middle satellite image contains the location of the shallow regional wellfield, developed in 1998 for the Integrated Water Supply Scheme (IWSS), and the location of the seawater intrusion monitoring (SIM) wells. The approximate annual groundwater abstraction from the local wellfield during peak production in 2002 was approximately 14 GL/year. The increase in groundwater abstraction during the monitoring period is one potential cause for seawater intrusion at the reference site. Map imagery ©2019 Google Earth, Maxar Technologies.

The recent growth of the Quinns Rocks urban footprint is shown in Fig. 7, and includes population and IWSS groundwater abstraction between 1984 and 2016. The local IWSS wellfield is approximately 2 km to 4 km from the coastline. Shallow groundwater abstraction from the Quinns Rocks region has supplied an average of 12 GL/year since 2003. This forms the majority of coastal groundwater abstraction and accounts for approximately 75% of local groundwater usage^42^.

The development of a groundwater abstraction wellfield parallel to the coast and increase in urban land usage around the reference site is a strong motivation to simulate the local groundwater hydraulics and hydrogeology. Although it may seem easy to attribute the data to groundwater abstraction alone, there are other factors that may have influenced the position of the seawater wedge at this site.

### Influence of a changing climate and reduced rainfall recharge

The impacts of a rapidly changing modern climate are well-documented^83–87^. Changes in rainfall patterns and seasonal temperatures will impact groundwater systems^88,89^. For example, the reduction in winter rainfall can reduce groundwater recharge to a shallow aquifer system, reducing aquifer flows, and ultimately resulting in seawater intrusion^90^. Bryan, et al^64^ suggest that declining rainfall in the Perth region is the primary cause of seawater intrusion for Rottnest Island, located just 20 km offshore from Perth.

**Figure 8:**
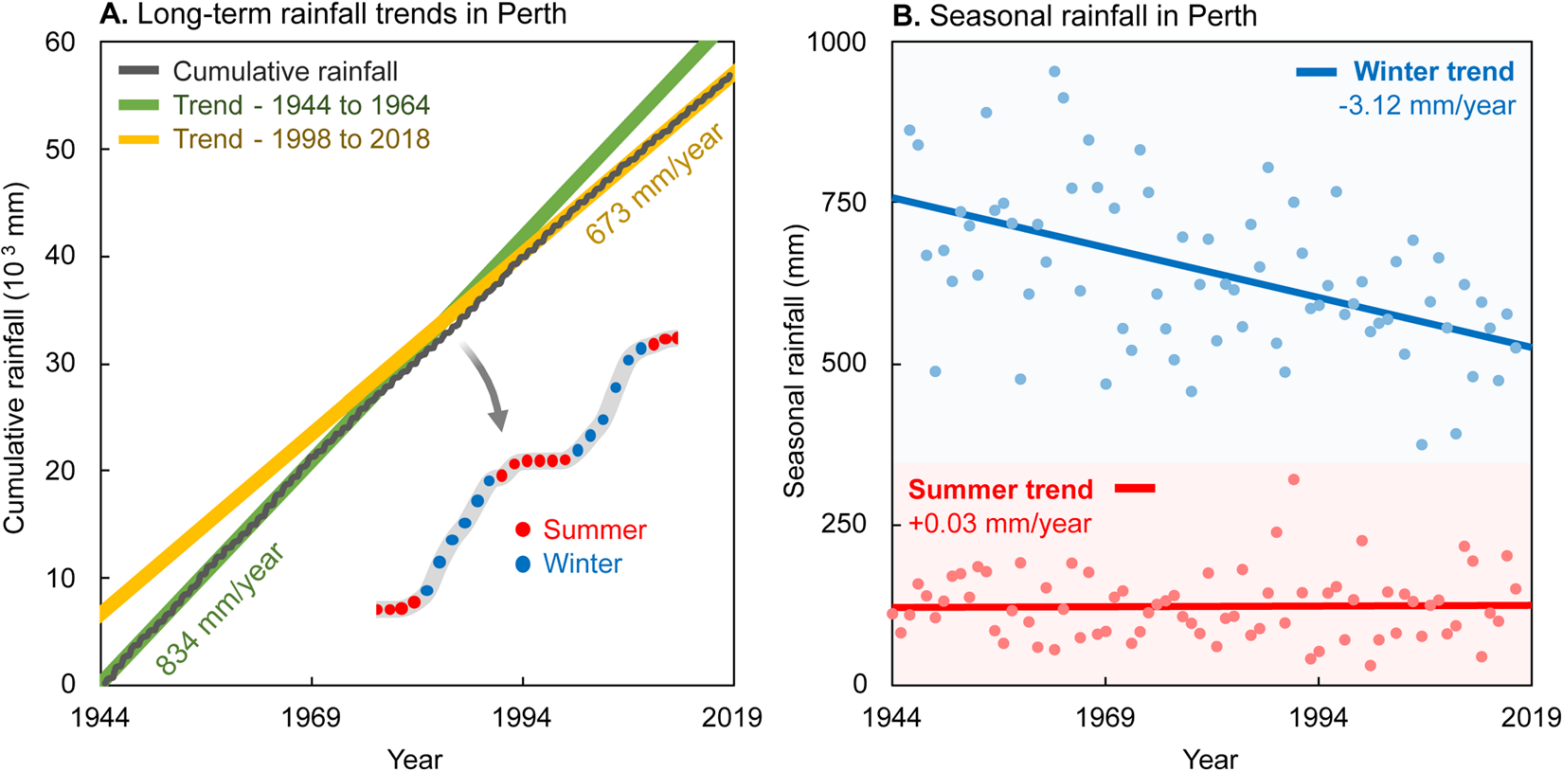
Graphs showing long-term trends in winter and summer rainfall in Perth, Western Australia. Panel A shows the cumulative rainfall from 1944 to 2018. The average rainfall over the entire period is approximately 700 mm/year. Analysis of 20-year trends indicate that an approximately 25% reduction in rainfall has occurred since the start of measurement in 1944. The average rainfall from 1944 to 1964 was 834 mm/year, compared to data from 1998 to 2018 with an average rainfall of 673 mm/year. Panel B shows the total seasonal rainfall for summer and winter showing that a clear decrease in winter rainfall. The decreasing winter rainfall is strong motivation for understanding the follow-on effects to the seawater interface.

Figure 8 shows the cumulative and seasonal (six-monthly) rainfall in Perth between 1944 and 2018. The long-term average rainfall is approximately 700 mm/year. A comparison of the first and last 20-years of the data suggests that the average rainfall has decreased from approximately 834 mm/year (1944 to 1964), to 673 mm/year (1998 to 2018).

Analysis of summer (October to March) and winter (April to September) rainfall trends suggest a minor increase of 0.03 mm/year in summer rainfall. However, groundwater recharge from summer rainfall is negligible due to high evapotranspiration rates in Perth^60,91^. Winter rainfall contributes to the vast majority of rainfall recharge. Analysis of rainfall trends from Fig. 8 suggests an approximately 25% reduction over the 20-year period between 1998 and 2018 compared with the 20-year period between 1944 and 1964.

### Influence of sea level rise

Changes in the global sea level, and sea level rise in particular, can affect the position of the seawater interface and the near-shore environment^61,92–94^. Simulations of the impact of sea level rise suggests that relatively small increases in sea level can potentially move the seawater interface landwards by hundreds of metres^95,96^. This can extend up to kilometres where karstic high-flow conduits are present^21,97^.

**Figure 9:**
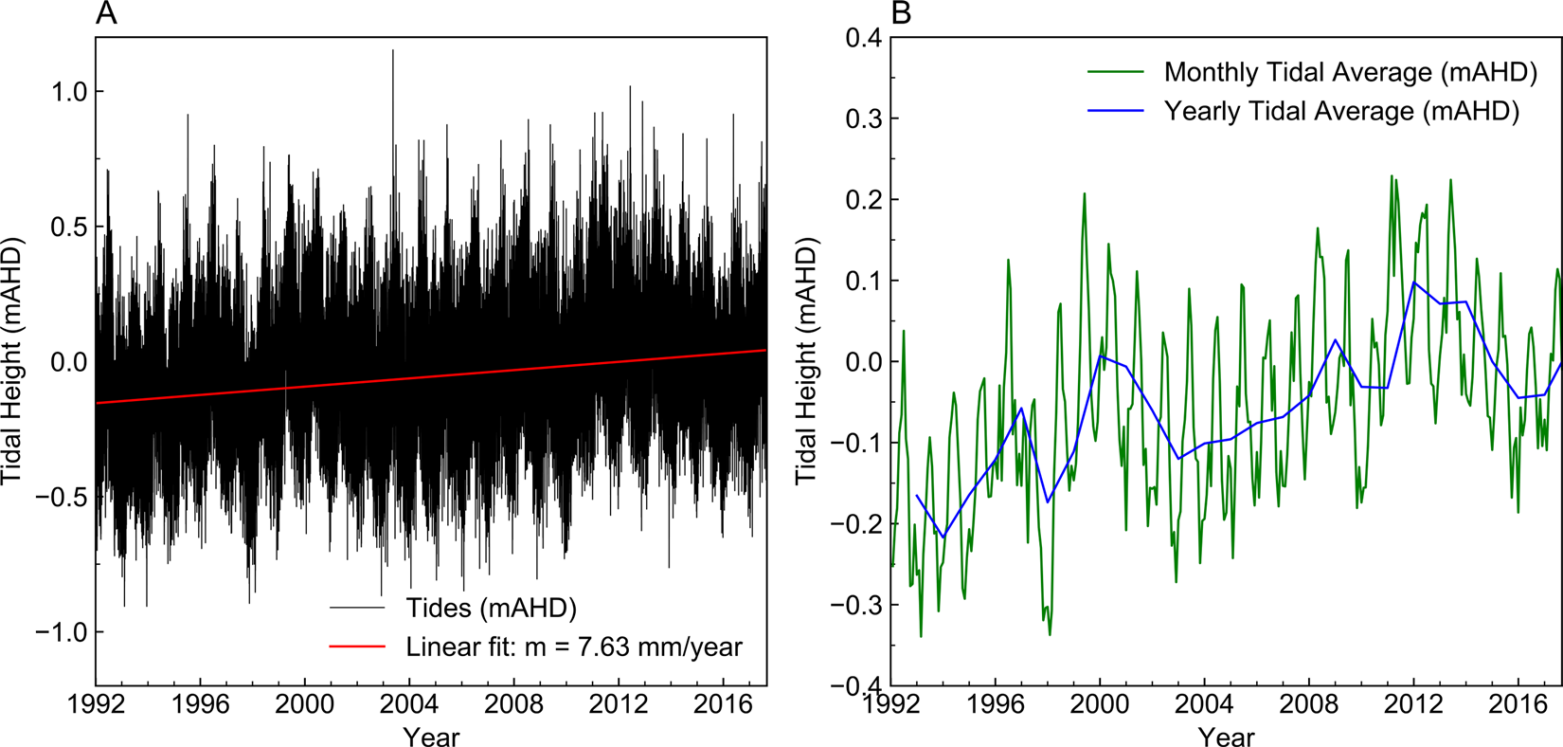
Charts showing tidal measurements from a monitoring location near the reference site in meters Australian height datum, mAHD. Panel A shows the raw hourly data since 1992 with a linear fit overlaid. Panel B shows the monthly and yearly averages of the raw data. Tidal levels have risen by approximately 7.63 mm/year since monitoring began. The estimate for sea-level rise that also considers global meteorological events is 9.0 mm/year^98^. The rise in sea level over the monitoring period is a potential factor for landward movement of the seawater interface at the reference site.

Measurements from a tidal monitoring station located approximately 2 km south of the Quinns Rocks reference site suggest the sea level, on average, has risen by 7.63 mm/year. This is shown in Fig. 9. The government estimate for sea-level rise, accounting for ENSO meteorological events, is 9.0 mm/year^98^. Sea level rise accounts for between 22.8 cm and 27 cm of increased seawater head at the coast over the 30 years of monitoring. The increase in mean sea level is a possible reason behind the observations at the reference site.

## Part 3. Predicting the Shape of the Seawater Interface: The Value and Limitations of Historical Monitoring Data

Monitoring wells provide data required to inform aquifer management. The quality and value of the information gathered is highly dependent on the design and placement of the monitoring well. The demand for the precise management of aquifer systems that works in conjunction with modern numerical groundwater modelling is outpacing both the data type and quality collected from existing monitoring networks.

**Figure 10:**
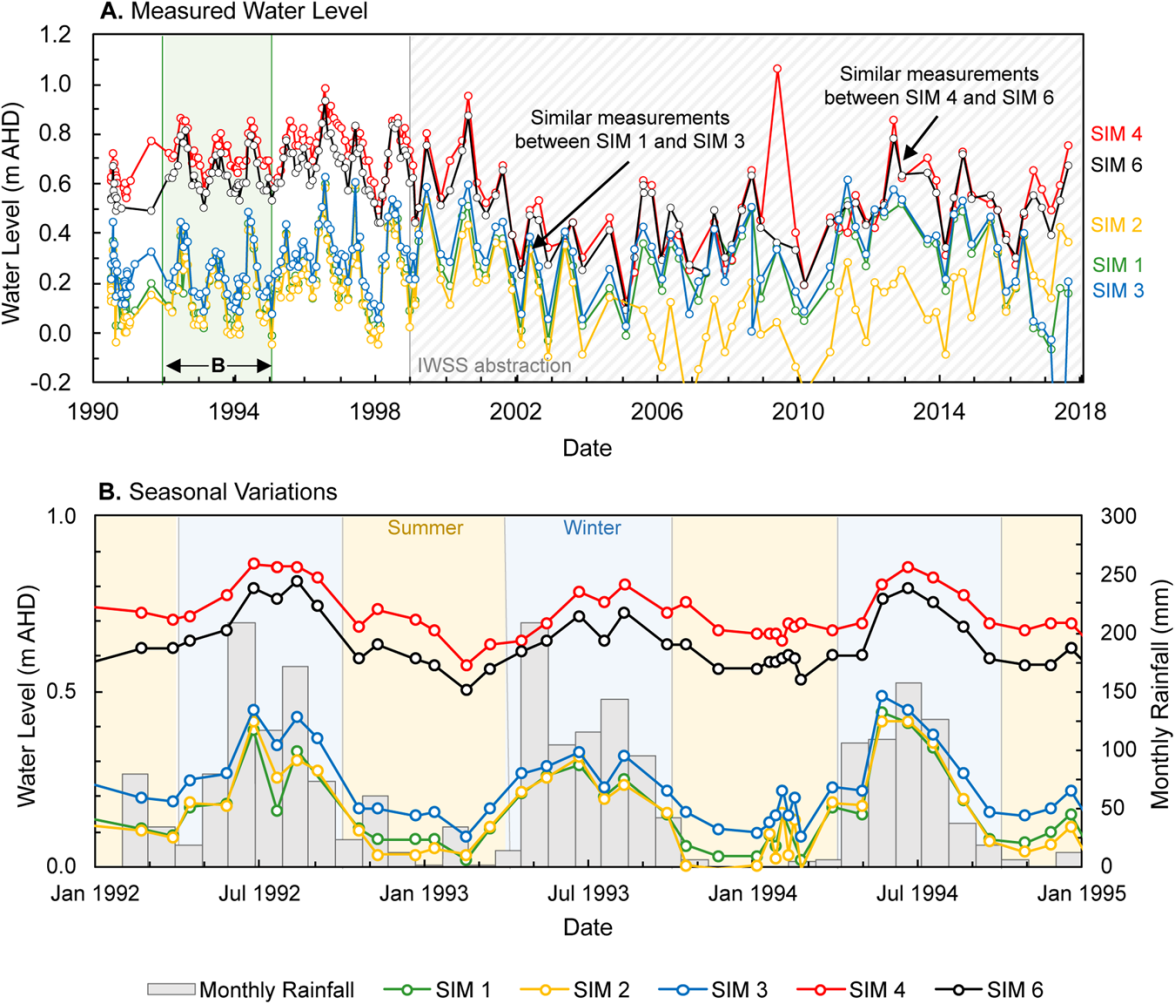
Set of graphs comparing measured water levels in the seawater intrusion monitoring (SIM) wells to monthly rainfall. Panel A shows measured water levels from 1990 to 2018. The frequency of measurements decreases after the IWSS pumping begins. The measured water levels in SIM 1, SIM 2, and SIM 3, are similar despite separation of 150 m (see Figs. 3 and 7). SIM 4 and SIM 6 also show similar measured water levels despite being separated by close to 200 m. Shallow gradients between these monitoring points suggests zones of localised extreme permeability. Panel B shows water levels and monthly rainfall between 1992 and 1995. Summer periods (October to March) are shaded orange and the winter periods (April to September) are shaded blue. A clear relationship between annual rainfall cycles and water levels is present.

Water level measurements in the seawater intrusion monitoring (SIM) wells at Quinns Rocks between 1990 and 2017 are shown in Fig. 10. Panel A shows distribution of measured water levels. The wells SIM 1 and SIM 3, nearest to the shoreline, have similar water measurements despite being approximately 80 m apart. The wells SIM 4 and SIM 6 are further inland and have similar measurements despite being over 200 m apart. The shallow hydraulic head gradient between these wells are consistent with a highly permeable aquifer. However, significant differences in hydraulic gradients measured between each pair of SIM wells also suggests a highly heterogeneous distribution of hydraulic parameters.

Panel B of Fig. 10 shows the variation in rainfall associated with the typical dry summer and wet winter cycles. The measured water levels are driven by seasonal rainfall cycles. In 1994, the water levels measured in SIM 2, approximately 30 m from the shoreline, vary between 0.00 mAHD (Australian height datum) during the summer months, up to 0.42 mAHD in the winter months. Daily tidal effects account for another 0.5-metre variation superimposed on the measured water levels.

Although there appears to be a substantial monitoring dataset at the Quinns Rocks reference site, we notice several inadequacies. Two key outcomes from analysis of the data in Fig. 10 are:

I. The temporal sampling rates from manual logging are unable to capture the response of the aquifer system from tidal variations, storm surges, and other rapid events (e.g. the spike in SIM 4 in 2009).
II. Measuring a single point in depth with conventional PVC wells could mask changes in hydraulic head or solute concentration due to the extreme variability associated with karstic aquifers (e.g. SIM 1, SIM 3 measurements and depths).

### Measurements of water chemistry - What range of seawater interface geometries fit monitoring data?

Sampling the properties of the groundwater, such as the electrical conductivity (EC), provides baseline data for identifying seawater intrusion. EC measurements can be approximated to a solute concentration (or total dissolved solids, TDS) using linear approximations^99^, or by more advanced approximations such as the equation of state of seawater EOS-80^100^. Details on these calculations as applied at Quinns Rocks are provided in Supplementary Table S6.

**Figure 11:**
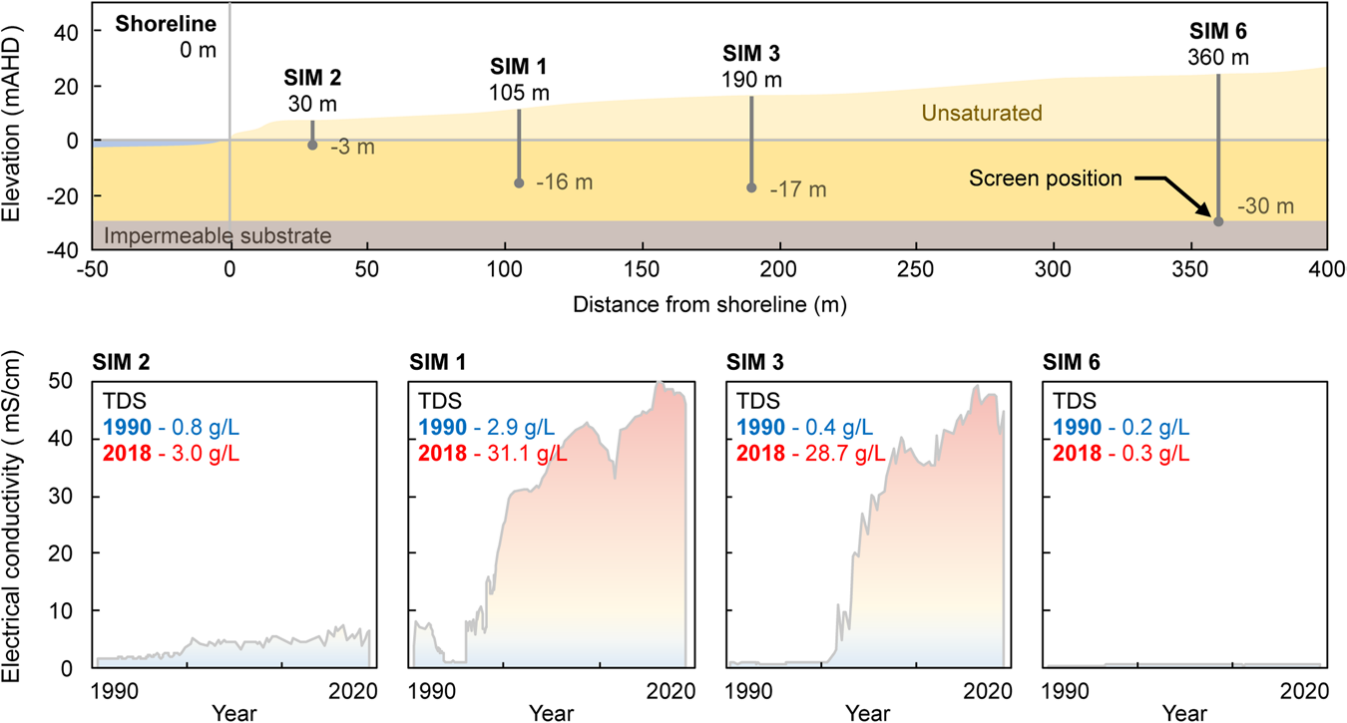
Cross-section through the SIM wells and accompanying time-lapse electrical conductivity (EC) data (1990–2018) showing evidence of seawater intrusion. The current position of the seawater interface is between SIM 3, where EC is equivalent to that of seawater in 2018, and SIM 6, where EC remains that of potable water in 2018.

Over the ~30 years of monitoring, EC data from the SIM wells clearly shows that movement of the seawater interface has occurred (see Fig. 11). The position of the interface in 2018 is somewhere between SIM 3 and SIM 6 (i.e. between 180 and 360 m from the shoreline). The solute concentration in SIM 3 is near to that of seawater at ~30 g/L, while in SIM 6 the groundwater has always remained fresh (i.e. potable), with a TDS of 0.3 g/L.

**Figure 12:**
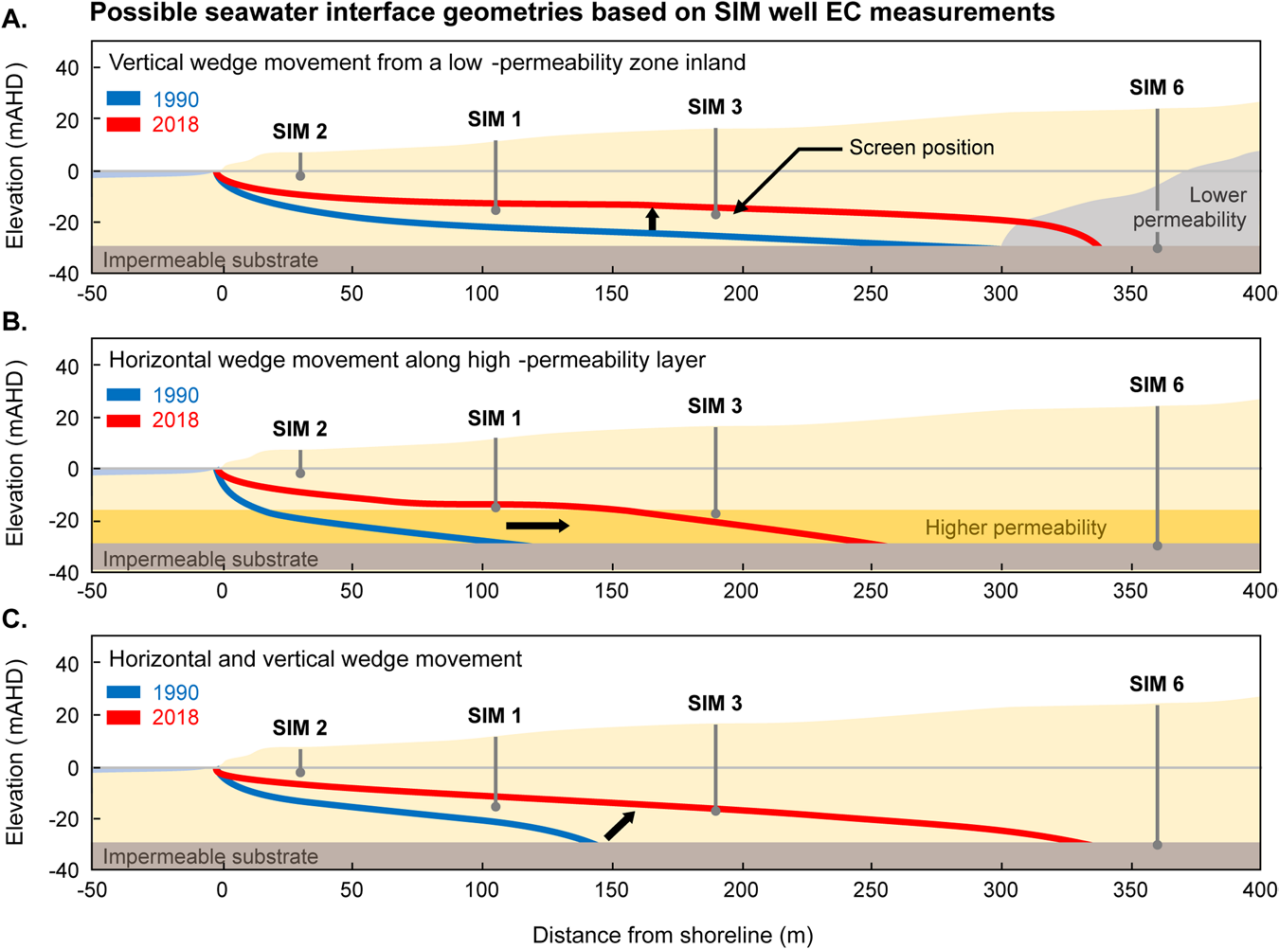
Schematic indicating the possible locations of the seawater interface based on measured solute concentrations from the SIM wells in 1990 (red line) and 2018 (blue line). Panel A shows an example of primarily vertical movement due to a zone of reduced permeability near to the toe. Panel B shows an example of horizontal movement of seawater, such as along a high-permeability conduit located near to the base of the aquifer. Panel C shows an example of both horizontal and vertical movement inland, as would be found in a homogeneous aquifer.

A disadvantage of electrical conductivity measurements made in the SIM wells is that the measurements are limited to a single short-screened interval (see Table 1 for the screened intervals in the SIM wells). This allows for many interpretations of the shape of the seawater interface, such as the three hypothetical scenarios that may all correspond to same EC measurements within the SIM wells (see Fig. 12). These scenarios are intended to highlight end members from a range of potential geometries of the seawater wedge over the monitoring period. They include:

**Scenario 1** (Fig. 12A): Here the toe remains relatively stationary while the seawater interface expands vertically. This extreme may occur where there exists an inclined substrate^65,101^, or an extremely low-permeability lithology exists near the toe^95,102^.

**Scenario 2** (Fig. 12B): The seawater wedge expands horizontally. This may occur if horizontal layers have extremely high horizontal hydraulic conductivity, such as directional preference conduit systems^21,38,103^.

**Scenario 3** (Fig. 12C): The seawater wedge expands both horizontally and vertically. This is perhaps the most commonly reported movement in seawater intrusion literature^2,17,63,104^.

Long-term groundwater monitoring at the Quinns Rocks reference site shows that movement of the seawater interface has occurred during the monitoring period and that the seawater interface currently exists between 200 m and 360 m from the shoreline. However, it is not possible to reconstruct the seawater interface or make conclusions concerning the rate of intrusion from this data alone.

### Measurements of hydraulics across the wedge transition

Measurement of the hydraulic head at the coastal margin can present greater uncertainty than in other groundwater systems^105^. These uncertainties can be driven by large variations in groundwater density and a dynamic groundwater environment. A key uncertainty from aquifers containing variable-density groundwater arises when the density of water in the well column is not constant or accurately known when the water level measurement is made.

It is often necessary to convert the measured water level in coastal wells to pressure expressed as an equivalent freshwater head for numerical modelling. In a static system, hydraulic head (${h}_{i}$) is the sum of elevation head (${z}_{i}$) (i.e. the depth of the well screen), and pressure head (${h}_{p,i}$) (i.e. the length of water column relative to ${z}_{i}$)^106^. This is described by

1 $$\begin{array}{c}{h}_{i}={z}_{i}+{h}_{p,i}={z}_{i}+\frac{{P}_{i}}{{\rho }_{i}g}\end{array}$$

where ${h}_{p,i}$ is the hydraulic head from pressure at point$\,i$, ${P}_{i}$ is the pressure at the well screen,$\,{\rho }_{i}$ is the fluid density of the groundwater at the well screen, and$\,g$ is the acceleration due to gravity.

If unknown variable groundwater density exists, the same hydraulic head (e.g., measured water level) could be interpreted from different hydraulic pressures. For a system with groundwater of varying density, the ‘equivalent freshwater head’^106,107^ represents the column of fresh groundwater required to balance the hydraulic pressure at a particular depth and groundwater density. The equivalent freshwater head for groundwater at point $I$ with density ${\rho }_{i}$ is:

2 $$\begin{array}{c}{h}_{f,i}=\frac{{\rho }_{i}}{{\rho }_{f}}{h}_{i}-\frac{{\rho }_{i}-{\rho }_{f}}{{\rho }_{f}}{z}_{i}\end{array}$$

where ${\rho }_{f}$ is the density of fresh groundwater.

**Figure 13:**
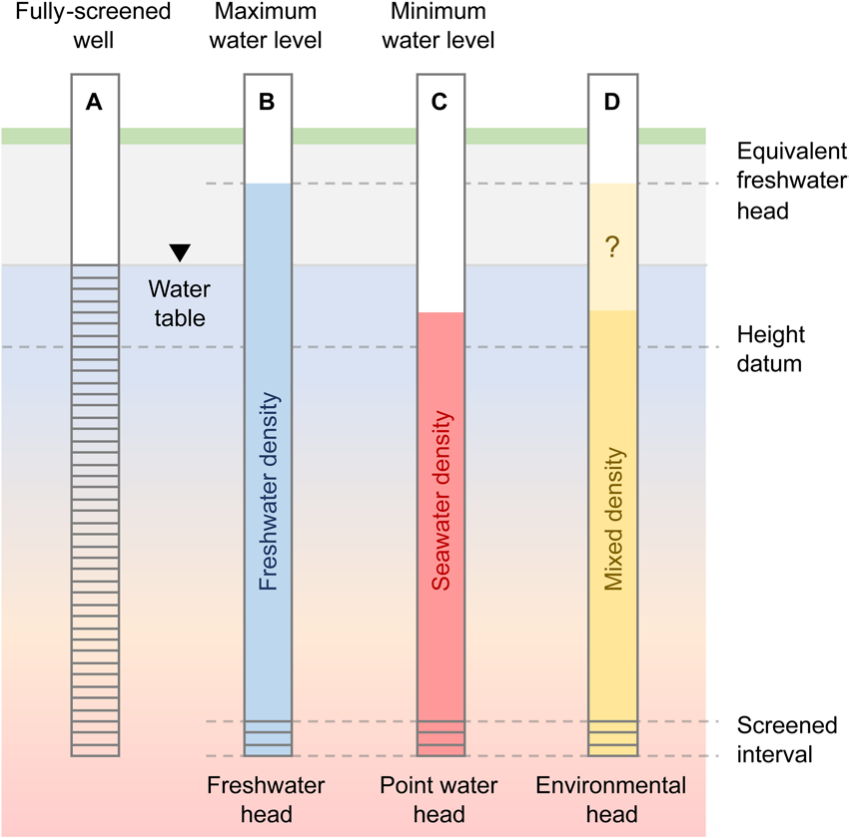
Schematic representation of uncertainty in the measurement of the hydraulic head from a well where large changes in solute concentration exist, such as at the seawater interface. The water level measured in a monitoring well is dependent on the density of water in the well column^106,107^. Well A is a fully screened well where the groundwater inside the well column matches that outside of the well. Here the measured water level is equivalent to the water table. Well B is screened below the water table. If fresh water occupies the entire water column, the measured water level will be above the water table. Well C shows that if the well column is filled with seawater, the measured water level will be below the water table. Well D assumes that the density of the water column is a mixture of fresh and saline waters. This observation is critical to compare measured water levels with numerical modelling outcomes, which are typically provided as pressure in equivalent freshwater head. If the water density in the well column is not measured at the time of the water level measurement, there is significant uncertainty in the equivalent hydraulic head calculation.

The range of densities for groundwater proximal to the seawater wedge can lead to multiple interpretations of hydraulic head. These are illustrated in Fig. 13.

1. If the well is fully screened across the aquifer, the measured water level is equal to the ‘*in situ*’ hydraulic head, as shown in Fig. 13A. However, fully-screened wells (Fig. 13, Well A) are susceptible to passive redistribution of groundwater along the screened interval between layers with different hydraulic properties^108^. Any vertical movement or redistribution of seawater via the well-column can affect the groundwater salinity measurements with consequence for interpretation and monitoring.
2. For the equivalent freshwater head (Fig. 13, Well B), the pressure at the screens is represented by the equivalent column of fresh water. The equivalent freshwater head is always higher than the measured water level when high-density groundwater is present.
3. The point-water head (Fig. 13, Well C) assumes that the groundwater density at the screened interval exists throughout the well column. If there is seawater at the screened interval, the measured water level inside of the well is lower than the true water level outside of the well. This could occur if the screened interval of a monitoring well is located within the seawater wedge and has been sampled through pumping.
4. The environmental head is calculated assuming that the water within the well is stagnant, and the density of water inside the well column is equal to the average of water outside of the well^106,109^ (see Fig. 13, Well D).

A consequence of the above is that the measured water level may not be a reliable input for computation of groundwater flow conditions in variable density environments. Here specific measurements are required to characterise these groundwater flow systems. We discuss suitable monitoring techniques in the conclusion.

**Figure 14:**
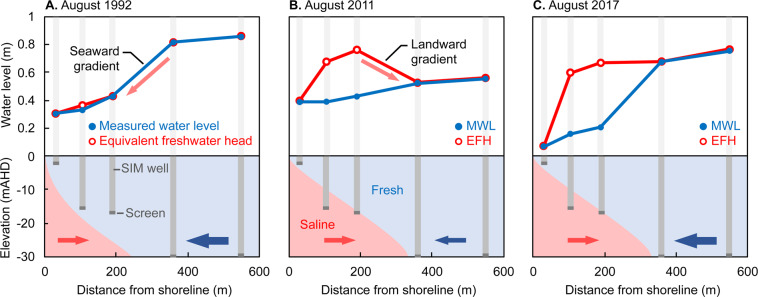
Charts and schematic showing the equivalent freshwater head compared to the measured water levels. Here the equivalent freshwater head (EFH) is calculated assuming that the water density around the well-screen exists throughout the column. Panel A shows a measurement from 1992, which indicates that fresh water is present. Here the hydraulic gradient is towards the ocean. Panel B shows a measurement from 2011, where EC measurements at the well-screens suggest that high density seawater is present. Panel C shows a measurement from 2017, where the EFH in SIM 6 is higher than in SIM 3, suggesting no landward movement of the seawater interface is occurring. The measured water levels in Panel B suggest a seaward hydraulic gradient, however the density of water in the well columns has not been considered. Estimation of the EFH with the assumption that seawater has filled the well column for SIM 1 and SIM 3 in 2011 and 2017 presents significant changes in groundwater hydraulics in this shallow coastal aquifer system.

The density of water residing in the well column is often not measured directly, and so must be assumed from the EC of water samples. This is the situation for the SIM wells at the Quinns Rocks reference site in Perth, Western Australia. Figure 14 provides an example from the reference site showing the difference in hydraulic head after computing the equivalent freshwater head (EFH) using the EC-derived mass density^100^, compared to the measured water level (MWL). The three dates shown cover the time before seawater intrusion (Fig. 14A), during active seawater intrusion (Fig. 14B) and a recent date where the EFH gradient is approximately seaward (Fig. 14C).

The well-to-well hydraulic gradients computed from the measured water levels (i.e. the blue line) in every date shown in Fig. 14 suggests that groundwater is flowing towards the ocean. In 1992 (Fig. 14A), all of the SIM wells contain relatively fresh groundwater and the equivalent freshwater head is similar to the MWL. However, by 2011 (Fig. 14B) seawater had progressed beyond the screened interval in SIM 1 and SIM 3 (see Fig. 11). The equivalent freshwater head suggests a landward hydraulic gradient between SIM 3 and SIM 6.

At first this may seem impossible or at least counterintuitive, however the water level measurements at SIM 1 and SIM 3 must be considered in the context of a seawater recirculation cell (see Fig. 2 and Part 6) that has potentially moved inland beyond the wells. This presents the possibility of landward flow, albeit at exceedingly low velocity, in SIM1 and SIM 3 compared with expected groundwater flow towards the ocean past the screen in SIM 6, which remains fresh.

### Estimation of groundwater throughflow from hydraulic gradients

Hydraulic gradients at coastal margins can be influenced by seasonal changes in groundwater recharge, sea-level variations, and groundwater abstraction (see sections above). Estimates of throughflow based on hydraulic gradients must also be affected by how gradients are computed. For example, the gradients are dependent on how aquifer pressure is calculated (i.e. freshwater head), and localised impacts on aquifer pressure, (e.g. tidal forces and groundwater abstraction).

We estimate groundwater throughflow to the ocean based on hydraulic gradients. The question being addressed is “Can these methods provide reasonable estimates of groundwater throughflow for calculation of the landward extent of the seawater interface in a high-quality coastal aquifer system, such as at the Quinns Rocks reference site”?

There are several assumptions made when estimating groundwater throughflow using the flow-nets and hydraulic gradients. A typical flow-net analysis assumes that a homogeneous, saturated, and isotropic aquifer with known boundaries exists^110^. Extensions to these assumptions exist for anisotropic aquifers^111^ and partially saturated flow systems^112^. The groundwater throughflow, *Q*, is typically estimated from^42^

3 $$\begin{array}{c}Q=TiL\end{array}$$

where *T* is the transmissivity (m²/day), *i* is the hydraulic gradient across the aquifer (m/m), and *L* is the width of the flow-cell (m). Here we assume the flow-cell is of unit length. Transmissivity (T) is the product of the hydraulic conductivity (K) (m/day) and the thickness of the freshwater saturated aquifer (i.e., where the fresh groundwater enters the system and occupies the aquifer thickness) (m). For the Quinns Rocks reference site, Kretschmer and Degens^42^ estimate the mean hydraulic conductivity to be 130–200 m/day, and the saturated thickness of the aquifer (i.e. depth to confining substrate) to be 30 m^42^.

**Figure 15:**
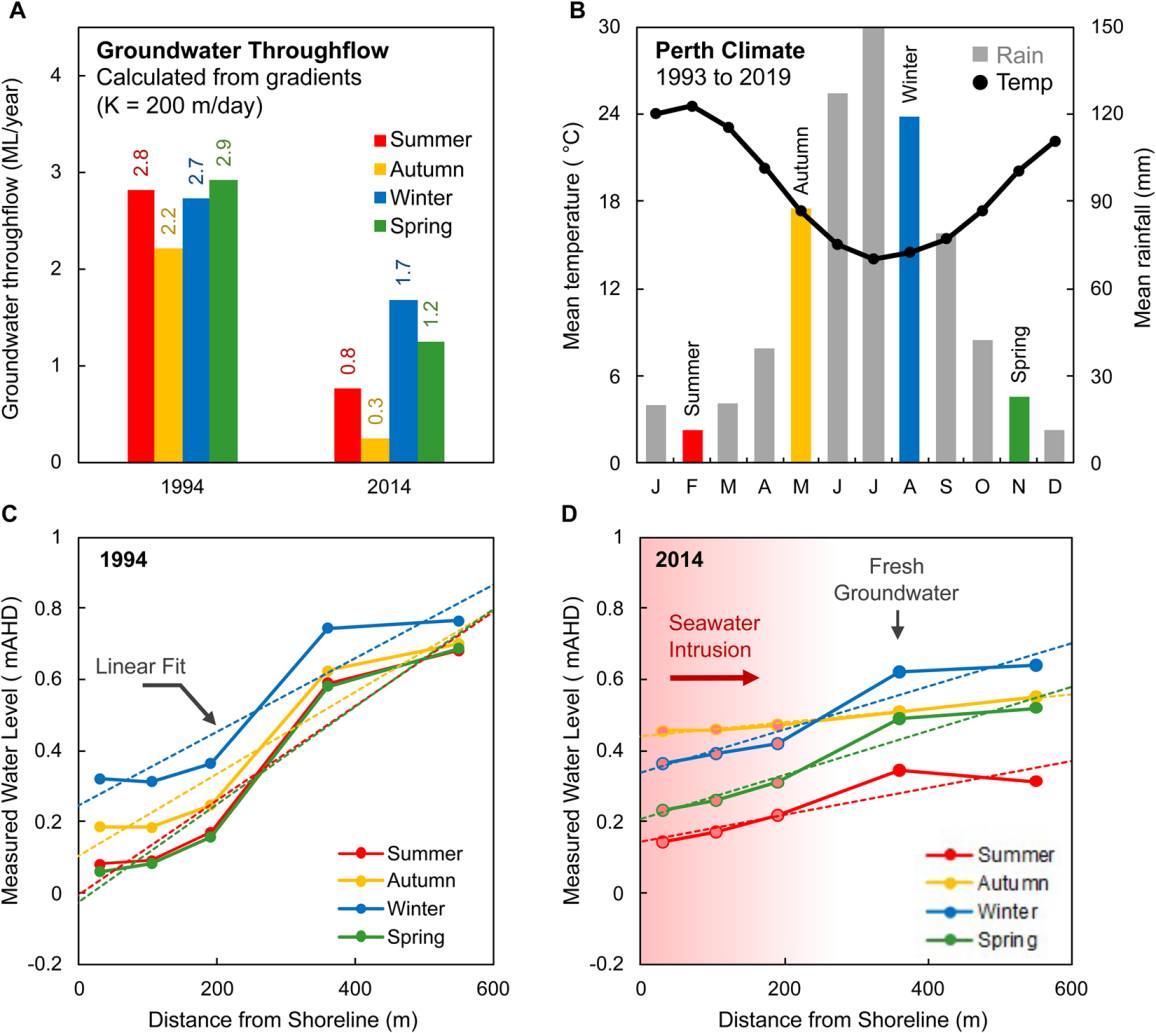
Set of charts showing seasonal variations in groundwater levels, climate and estimated throughflow at Quinns Rocks. Panel A shows the groundwater throughflow, Q, calculated from hydraulic gradients using hydraulic conductivity K = 200 m/day. Gradients are computed from the raw measured water levels^188^. Panel B shows the mean seasonal temperature and rainfall between 1993 and 2019. Panels C,D show the average seasonal groundwater levels measured in the SIM wells along with estimated linear fits for 1994 and 2014 respectively. The average groundwater throughflow during 1994 is significantly higher than in 2014, however we must acknowledge that there are significant uncertainties in the inputs to estimates of hydraulic gradient and calculation of groundwater throughflow (see also Part 1.3, 3.2, and Fig. 14). At best these methods provide a first-order approximation of groundwater flow.

Two sets of estimates for the groundwater throughflow (Q) calculated from the hydraulic gradients of the measured water levels across the SIM wells are shown in Fig. 15. This includes estimates for 1994, prior to regional groundwater abstraction, and in 2014 after seawater intrusion has occurred. The average groundwater throughflow is estimated to be 3 ML/year and 1 ML/year respectively. However, it is important to acknowledge the significant uncertainties that exist in the inputs to these equations, such as the impact of variable density groundwater on the hydraulic gradient, and the role of heterogeneous hydrogeology on transmissivity.

If EC measurements (and thus some estimate of density) are not made simultaneously with the measurement of water level, there may be no indication whether the measurement of hydraulic head (and thus the flow from hydraulic gradient) is affected by the impassable seawater wedge. It may seem appropriate to estimate the gradient from measurements in wells that are known to be fresh throughout the entire thickness of the aquifer, such as SIM 4 and SIM 6. As shown in Fig. 15, the hydraulic gradients between these two wells are significantly different to the hydraulic gradient taken across all five SIM wells and yield a far lower estimate of groundwater throughflow (see Supplementary Figures S1 and S2).

The hydraulic gradients between each of the SIM wells also questions the assumption of homogeneity for the transmissivity estimate. The steep gradient between SIM 3 and SIM 6 suggests a zone of lower hydraulic conductivity between the screened intervals of these wells. Shallower inter-well gradients, such as between SIM 1, SIM 2 and SIM 3, can be indicative of high hydraulic conductivity zones. Variable hydraulic gradients across the SIM wells provides evidence that the aquifer is heterogeneous.

At best, these methods provide a first-order approximation of groundwater flow. Groundwater flow has reduced from 1994 to 2014, however, the precise value of the groundwater throughflow is uncertain. We will see that combination of water level measurements and the simple methods described above cannot provide certainty for the landward extent of the seawater interface or groundwater throughflow.

### The seawater interface according to an analytical solution

Analytical solutions, such as those of Bear and Dagan^113^, Glover^18^, Strack^114^, and others^115^, estimate the position of the seawater interface based on averaged measures of hydraulics (e.g. the groundwater throughflow and average hydraulic conductivity). These solutions tend to simplify the transition between saline and fresh water to a sharp boundary, neglecting the effect of solute transport phenomena such as dispersion. They can have value in regional scale seawater intrusion studies where numerical modelling may not be practical^71^.

The Glover solution is used to illustrate the range of possible seawater toe positions that may be derived using data at the field site with constraints from groundwater throughflow estimates. The Glover solution is a readily applied analytical solution that is routinely used to estimate the steady-state toe position of a seawater wedge. It is expressed as^18^:

4 $$\begin{array}{c}{z}^{2}=\frac{2Q}{K\Delta s}x+{\left(\frac{Q}{K\Delta s}\right)}^{2}\end{array}$$

here *z* is the depth below sea level (e.g. 0 m) to the seawater interface (m), *Q* is the flow per unit length of the shoreline (m²/day), *K* is the hydraulic conductivity of the aquifer (m/day), Δ*s* is the density ratio of seawater to fresh water, and *x* is the horizontal distance inland from the shoreline (m).

The range of estimated groundwater throughflow from hydraulic gradient analysis using the average measured water level across all of the SIM wells is between 3.00 ML/year and 0.48 ML/year. At the lowest estimate of flow and using a hydraulic conductivity of 200 m/day, the Glover solution places the seawater interface at 1813 m inland from the shoreline. This estimate is not reasonable for the study site as fresh groundwater is still present at the SIM 6 monitoring well located only 360 m inland. We suspect that the gross overestimate (1813 m) for the position of the seawater interface is likely due to the inability of the solution to accommodate hydraulic complexity of the karstic system along Perth’s coastal margins, although there are many uncertainties in the inputs to this solution as discussed in 3.3.

In the next section, we use numerical solute transport models for a homogeneous aquifer to simulate the position of the seawater toe for comparison with the toe position calculated from the analytical solution and estimated from the field data.

### The seawater interface according to a homogeneous numerical transport modelling

Numerical groundwater flow and solute transport modelling can replicate phenomena observed at the seawater interface, such as groundwater mixing and variable density heads. We use FEFLOW 7.2^104^ to simulate seawater intrusion into a high quality, high permeability coastal aquifer similar to that found at the Quinns Rocks reference site. We use a 2D cross-sectional model to describe groundwater throughflow to the ocean using units of ML/year for a unit thickness (1 m). That is, for an aquifer that is 30 m thick, the groundwater throughflow is the rate that water passes through a surface with dimensions 1 m × 30 m.

**Figure 16:**
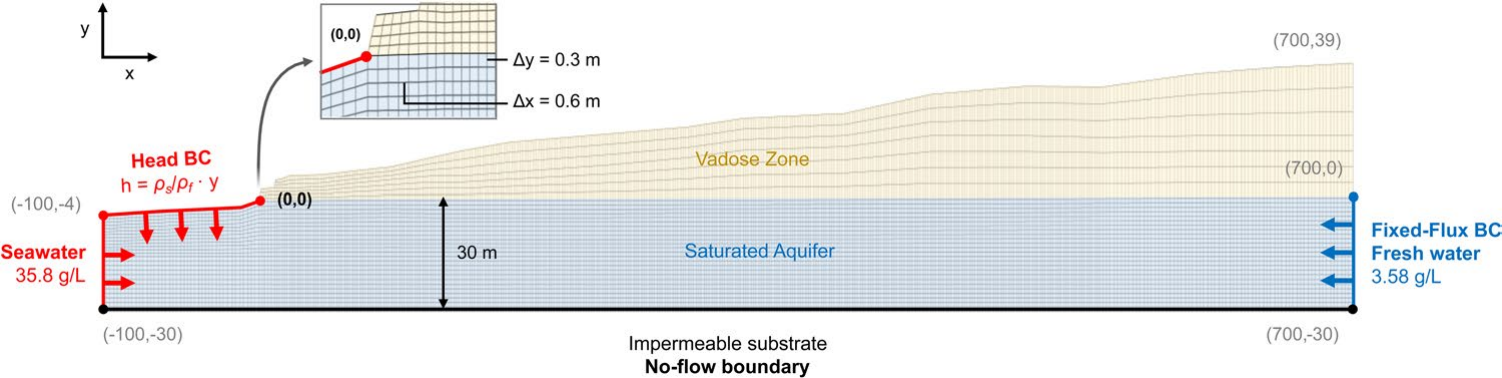
Diagram representing the finite element mesh and boundary conditions used for numerical groundwater flow and solute transport modelling. The mesh is coarse above the saturated zone as no recharge from the surface is included. Cells surrounding to the water table are included in the aquifer mesh refinement. The impact of increasing levels of refinement (e.g. number of elements) are provided in Supplementary Figure S3.

The quadrilateral mesh discretisation and boundary conditions used for the finite element model are shown in Fig. 16. Seawater enters the model through the left boundary and along the top boundary until the coastline (x ≤ 0 m). The density dependent hydraulic head condition ensures that seawater drives inland^116^. The flow of fresh groundwater (TDS ~358 mg/L) into the model is controlled by a fixed flux condition along the boundary at the right for *z* ≤ 0 m. The substrate at the base of the model (*z* = −30 m) represents a no-flow boundary.

Table 2 summarises the material parameters used in the groundwater flow and solute transport model. Simulations were run until no further changes to the position of the seawater toe were observed. The minimum mass concentration values are described in Part 1, while the maximum mass concentration is estimated from the sea-surface salinity local to Perth^117^. The density ratio is set to 0.0256 after Fofonoff and Millard Jr^118^. The dispersivity is estimated based on Smith, *et al*.^31^, Narayan, *et al*.^119^.

**Table 2:** Groundwater flow and solute transport modelling parameters used to generate models in FEFLOW.

Parameter	Value	Unit
Hydraulic Conductivity	200	*m*/*day*
Seawater Concentration (TDS)	35800	mg/L
Freshwater Concentration (TDS)	358	mg/L
Density Ratio	0.0256	—
Specific Storage	10^−4^	1/*m*
Effective Porosity	0.3	—
Molecular Diffusion	10^−9^	*m*^2^/*s*
Longitudinal Dispersivity	2	*m*
Transverse Dispersivity	0.2	*m*
Saturated thickness	30	*m*

**Figure 17:**
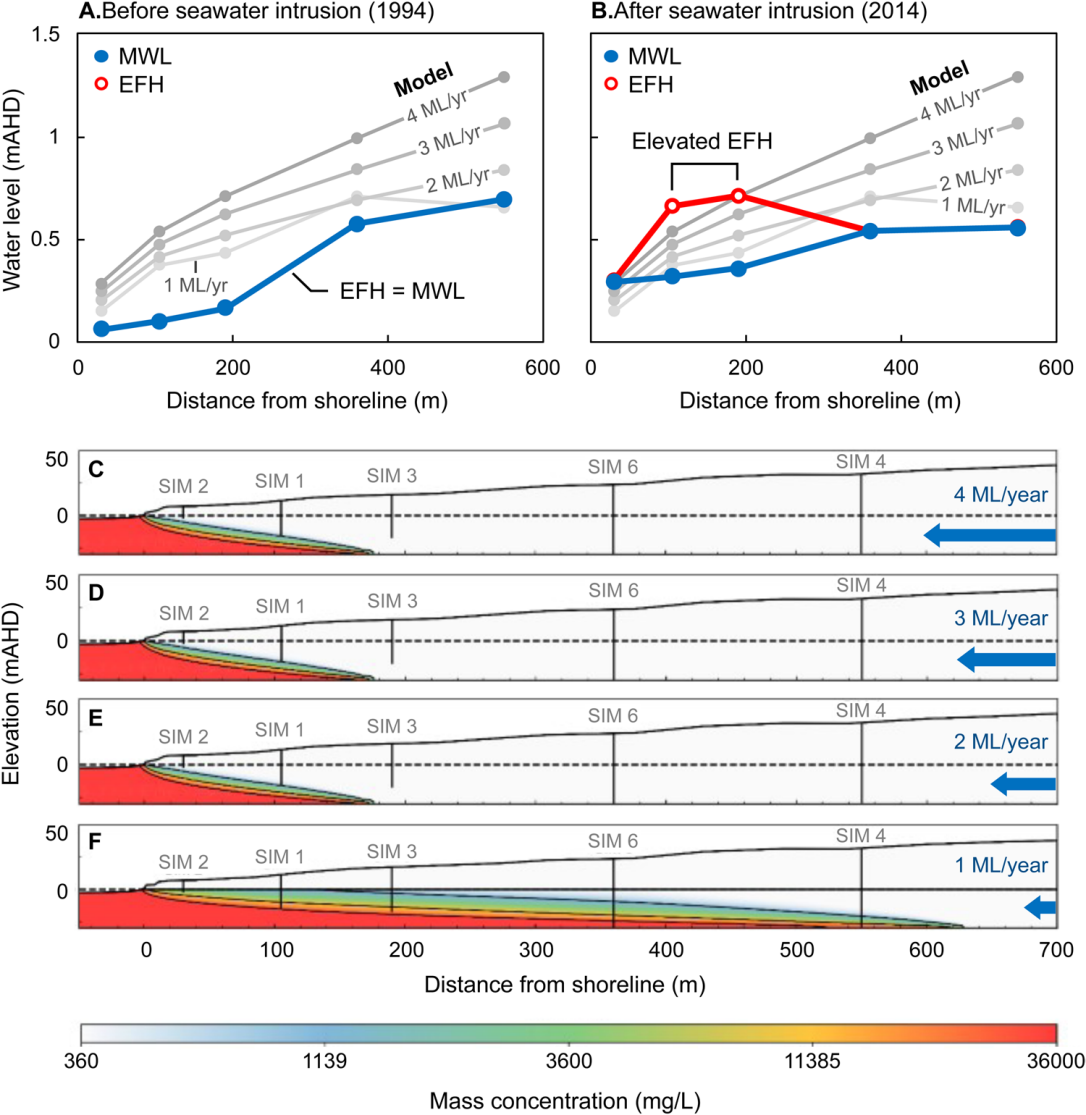
Set of images showing the simulated seawater interfaces for a range of groundwater throughflow in a 30 m thick aquifer with hydraulic conductivity 200 m/day over an impermeable substrate (i.e., average values for the Quinns Rocks reference site). Charts A and B show the measured water levels (MWL) and equivalent freshwater head (EFH) in 1994 and 2014. The EFH is calculated assuming that the groundwater at the well screen occupies the entire well column. Images C, D, E and F show the solute concentration distribution corresponding to groundwater throughflow of 4, 3, 2, and 1 ML/year respectively. According to this homogeneous aquifer model, groundwater throughflow must remain above 2 ML/year at Quinns Rocks to maintain fresh groundwater at SIM 6; however, this results in significantly greater simulated hydraulic head than the field observations. We find that there is no combination of hydraulic conductivity and throughflow for a homogeneous aquifer that can reasonably explain both measured values of hydraulic head and solute concentration at the reference site. This points towards high contrast in hydraulic parameters within the aquifer as a strong influence on the landward extent of saline groundwater.

We simulate the systematic reduction of groundwater throughflow and show the change in solute distribution in Fig. 17. The groundwater throughflow is reduced from 4 ML/year to 1 ML/year and covers the range of groundwater throughflow estimated for the Quinns Rocks reference site. The groundwater throughflow is not reduced again once the SIM wells furthest inland become salinised. Figure 17C–F shows that halving the groundwater throughflow will double the inland position of the toe for the homogeneous aquifer.

A comparison of the simulated data with measured field data from 1994 and 2014 (Fig. 17A,B) shows that no throughflow rate can be combined with a homogeneous aquifer to achieve a suitable match. The EC in SIM 6 is the strongest constraint for the inland position of the seawater wedge, which, as of 2019 remains that of fresh water. To satisfy the solute concentration condition at SIM 6 with this homogeneous aquifer model, groundwater throughflow must remain above 2 ML/year, which results in the simulated hydraulic head being significantly higher than any of the measured values. This numerical experiment also highlights the counterintuitive shape of the simulated hydraulic gradients. As denser seawater passes the monitoring well screen, the equivalent freshwater head can rise above the hydraulic head for wells further inland (in fresher water). This is shown by the hydraulic gradient between SIM 6 and SIM 4 (Fig. 17A,B).

### Comparison of numerical and analytic solutions for the landward extent of seawater

The landward extent of the seawater interface is fundamental information required coastal groundwater resource management. Although the analytical solution can provide a quick answer, we suspect that in practice it may also lead to significant error.

**Figure 18:**
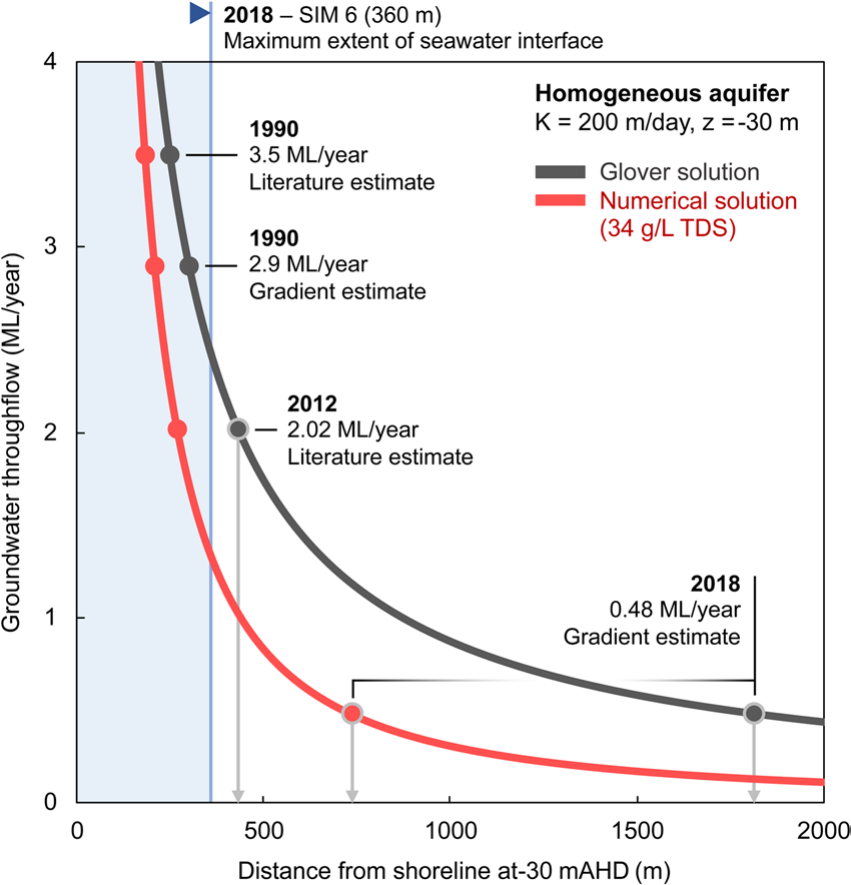
Chart comparing the landward position of the toe of the seawater wedge estimated from the Glover analytical solution (dark grey curve) and numerical simulations based on a homogeneous aquifer (red curve). We also provide a set of discrete throughflow estimates for several dates at the Quinns Rocks site to compare where these methods place the landward extent of the seawater wedge toe. These discrete groundwater throughflow estimations are derived from Kretschmer and Degens^42^ and hydraulic gradient analysis . Note that the seawater interface can be no more than 360 m inland (i.e. within the blue area) as electrical conductivity measurements in SIM 6 is screened at the base of the aquifer (see Fig. 11) and remains fresh. Both methodologies significantly overestimate the landward extent of the seawater interface at Quinns Rocks. We also note the significant difference between the toe positions estimated for the lower flow rate using the numerical solution compared and that estimated from the Glover solutions (see grey arrows marked on the image).

In Fig. 18, we compare the landward extent of the seawater toe using the analytical Glover solution with numerical simulations (see Table 2). Numerical modelling with throughflow of 1 ML/year places the toe at 434 m inland (i.e. this is the landward extent of the 34 g/L contour). For the same groundwater throughflow, the Glover solution places the toe at 875 m inland. This is almost twice as far inland as compared to the numerical model (see Fig. 17F). For a homogeneous aquifer, there is a significant difference between analytical and numerical solutions.

If we accept the simplifying assumption of a homogeneous aquifer for the Quinns Rock site, the numerical solution suggests that the reduction in groundwater throughflow between 1990 and 2018 should result in the seawater wedge moving over 500 m inland, to 750 m from the shoreline. This cannot be correct, as the groundwater in SIM 6, ~360 m from the ocean, has always remained fresh. This supports the case that neither the analytical solution nor numerical modelling with a homogeneous aquifer can reasonably estimate the landward extent of the seawater interface at Quinns Rocks. That is, no simplified model can reasonably match the measured water levels and solute concentration measured at the Quinns Rocks reference site.

The above outcome has practical consequences. As an example, approximately 23% of Western Australian households have a private shallow well used for household consumption or irrigation^120^. For the approximately 7,000 households local to the Quinns Rocks area^121^, approximately half of the dwellings are within 1 km of the coast. Over 800 shallow wells are potentially affected by groundwater management decisions at this relatively short interval of Perth’s coastal margin.

We suspect that numerical modelling with greater levels of complexity is required; however, this leads to a new question: “What data are required to build and constrain such numerical models?”. Below we consider the impact of anisotropy and strong hydraulic heterogeneity on the shape and landward extent of the seawater interface.

## Part 4. Complex Models: What is the Role of Anisotropy and Heterogeneity?

### How does anisotropy of hydraulic conductivity impact the landward extent of the seawater interface?

**Figure 19:**
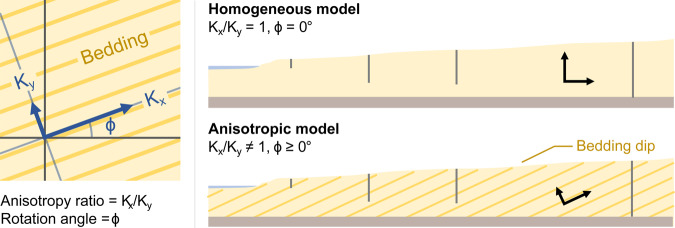
Schematic showing the ratio of anisotropy, *K*_*x*_*/K*_*y*_, in relation to the dip angle ($\phi$) for inclined bedding planes. Dune structures have dips that are typically 20° to 25°, with some aeolian systems having dips of 30° to 35°^125^.

The hydraulic conductivity in most sedimentary environments is likely to be anisotropic^122,123^ and must influence groundwater flow patterns in some way. Anisotropy can be introduced by grain-size variations from depositional cycles, crossbedding from dune cementation and limestone diagenesis^124^. For example, dune structures have dips that are typically 20° to 25°, with some aeolian systems having dips of 30° to 35°^125^. Anisotropy is expressed as the ratio of hydraulic conductivity, ${K}_{x}$/${K}_{y}$, where components of the conductivity tensor (${\boldsymbol{K}}$) are rotated by an angle ($\phi$)^104^ as shown in Fig. 19.

Anisotropy of hydraulic parameters can significantly impact on seawater intrusion^126,127^. Abarca, *et al*.^128^ and Kerrou and Renard^129^ use a dispersive anisotropic Henry problem^130^ to show that the penetration of the toe is predominantly controlled by the horizontal permeability (i.e., in the direction of flow) and geometric mean of dispersivity. Qu, *et al*.^131^ and Michael, *et al*.^132^ show that the anisotropic ratio (${K}_{x}$/${K}_{y}$) and position of the seawater interface are directly proportional.

**Figure 20:**
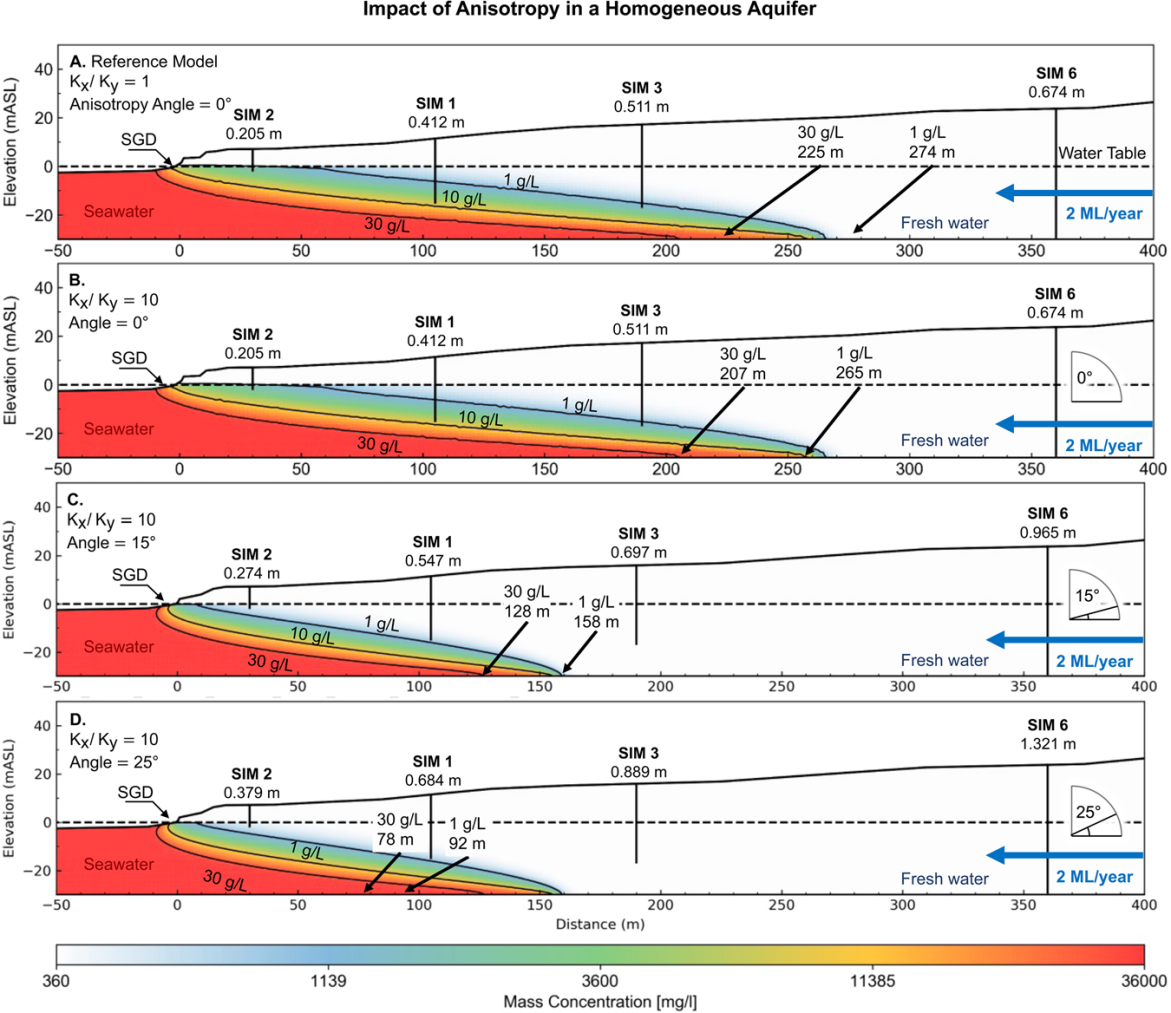
Images showing the influence of increasing anisotropic angle on the seawater wedge geometry in a high hydraulic conductivity (200 m/day) homogeneous aquifer. In this example, the angles of anisotropy are 0° (**B**), 15° (**C**), and 25° (**D**) degrees for a constant anisotropic ratio *K*_*x*_*/K*_*y*_ = 10. Panels A and B compare the isotropic and anisotropic models. The increasing angle of anisotropy is associated with higher hydraulic heads (annotated below each well), which is likely to be the primary driver behind the seaward movement of the seawater interface. The seawater wedge geometry in Panel D resembles the seawater interface geometry for a homogeneous isotropic model with a groundwater throughflow rate of 4 ML/year (see Fig. 15). This demonstrates that knowing the position of the wedge toe is not a reliable indicator of throughflow and vice versa.

We have found no examples that approach the influence of dip angle and anisotropy on the position of the seawater interface. We systematically demonstrate the impact of increasing the angle of anisotropy on the seawater interface geometry for a groundwater throughflow of 2 ML/year (see Fig. 20). The first model (Fig. 20A) is included for comparison with our baseline 30 m thick isotropic layer. Models for Fig. 20B–D, have the anisotropic ratio set to *K*_*x*_*/K*_*y*_ = 10, and a rotation angle of$\,\phi =0^\circ$, 15° and 25° respectively.

An interesting observation is that the zone of submarine groundwater discharge for the anisotropic model (Fig. 20B) extends further beneath the ocean than in the isotropic model (Fig. 20A), despite having the toe of the seawater interface located in the same position (~270 m). Qu, *et al*.^131^ suggest that the discharging zone must widen in order to maintain the discharge capability of the aquifer, with net seaward movement of the seawater toe. We observe this to a small extent in Fig. 20B, where the toe moves inland from 274 m to 265 m. Quantifying the impact of anisotropy on the extent of the submarine groundwater discharge may have practical implications for near-shore coastal ecosystems that are reliant on nutrient flow from terrestrial groundwater^4,6,133,134^.

A key finding expressed in Fig. 20, is that dip angle—regardless of the geological origin—has the potential to create hydraulic conductivity anisotropy and thus will also impact the landward extent of seawater at coastal margins. In Fig. 20, all examples use identical throughflow of 2 ML/year; however, the toe of the seawater interface in this otherwise homogeneous aquifer resides at 274 m, compared to 92 m for an anisotropy of *K*_*x*_*/K*_*y*_ = 10 at a rotation angle of 25°. This significant difference demonstrates the potential impact that anisotropy can have on the landward extent of the seawater wedge.

A more general observation is that the shape of the interface may not be a reliable mechanism for constraining groundwater throughflow and vice versa. We test this idea further below by manipulating throughflow and hydraulic conductivity anisotropy to achieve a constant hydraulic gradient.

### Is the position of the wedge a reliable indicator of groundwater throughflow?

**Figure 21:**
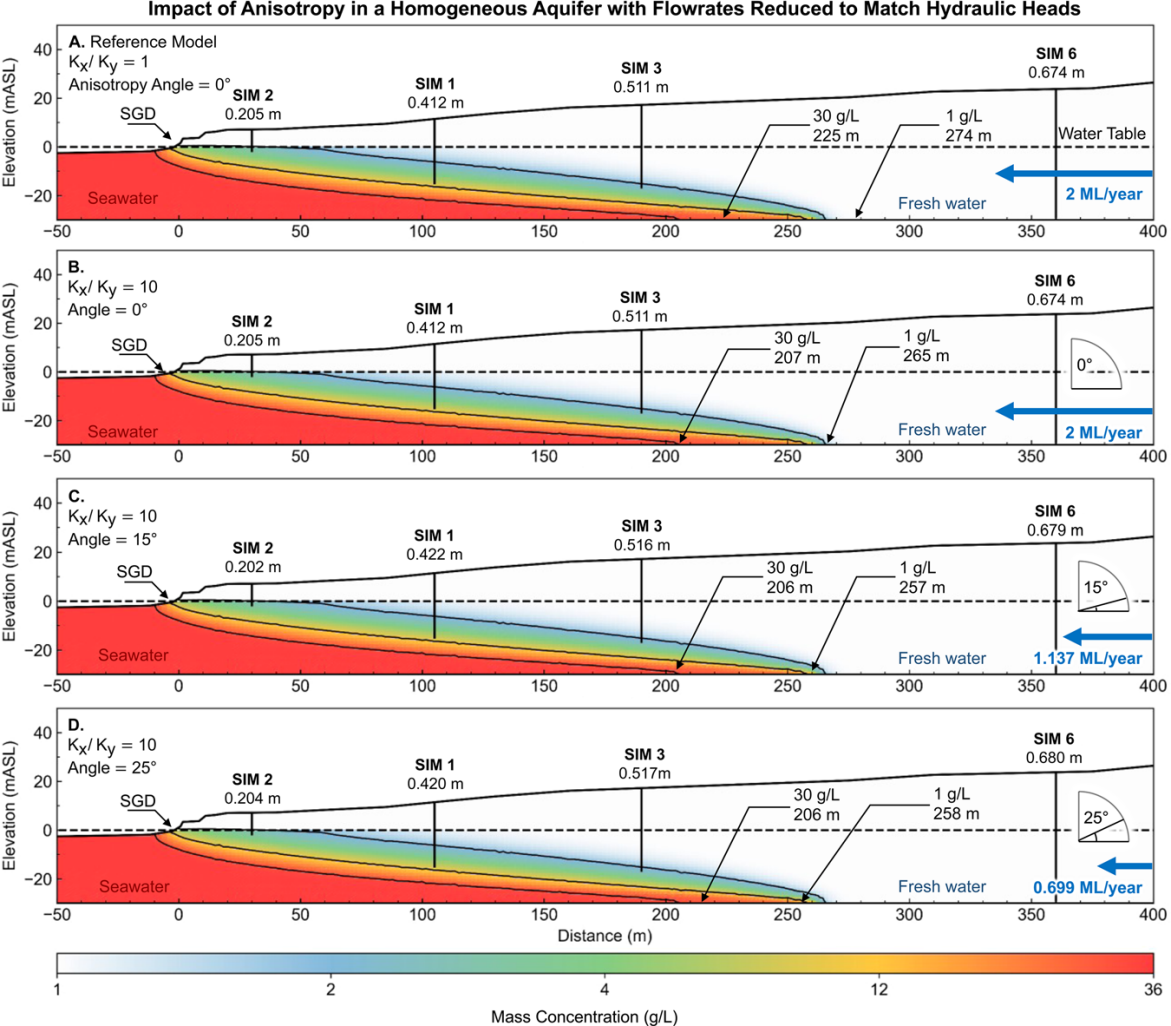
Images showing the influence of anisotropy on the seawater wedge geometry after reducing groundwater throughflow to match hydraulic heads. The differences between the resulting seawater wedge geometry is minor. For example, the wedge geometry from the lowest flowrate (0.69 ML/year) with anisotropic angle of 25° (Panel D) is similar to the wedge geometry at high 2 ML/year with an angle of 0° (Panel A). Lower anisotropic angles result in a wider zone of submarine groundwater discharge. This figure highlights the fact that knowing the seawater wedge position alone is not an indicator for groundwater throughflow. There is a clear need for better constraints on hydraulic parameters to understand the seawater interface in these coastal aquifers.

Hydraulic head is often used to constrain groundwater models. For the seawater interface, the hydraulic head gradients can be a key input to estimating throughflow to the ocean. However, the strength of this constraint in the absence of specific information regarding the hydraulic conductivity distribution needs to be carefully examined. We illustrate this point by taking a baseline scenario with hydraulic heads for a homogeneous aquifer (Fig. 21A) and then search for combinations of hydraulic conductivity anisotropy and groundwater throughflow that generates near-identical hydraulic head distribution. We present the results as the set of images in Fig. 21A through to Fig. 21D. By systematically increasing rotation angle for hydraulic conductivity anisotropy and decreasing throughflow, we maintain a constant hydraulic head distribution.

An additional observation from Fig. 21 is that the geometry of the seawater wedge at low throughflow rates (e.g. 0.699 ML/year, Fig. 21D) can be similar to the geometry of the wedge at much higher throughflow (e.g. 2 ML/year, Fig. 21A) by changing the hydraulic anisotropy rotation angle and or ratio. As a result, neither the groundwater throughflow estimation, nor the position of the seawater toe are reliable indicators of seawater intrusion in the absence of reliable and accurate information concerning hydraulic conductivity distribution or groundwater flow distribution.

In the next section, we consider spatially correlated random fields as a final higher level of model complexity and compare results to field data from the Quinns Rocks reference site. One reason for providing simulations with pseudo-random distributions of hydraulic properties is to examine the implications and limitations of monitoring well design (e.g. the SIM wells). For example, this provides a platform to assess the impacts of hydraulically connected high-permeability pathways (i.e. conduits or bedding planes) on measurement from a small number of wells.

### Taking model complexity to extremes: Spatially correlated random fields

A heterogeneous hydraulic conductivity distribution can be constructed with spatially correlated random fields^135^. Spatially correlated random fields can be used to generate geologically plausible distributions of subsurface parameters, such as hydraulic conductivity, porosity and dispersivity^122,136,137^. Each of these factors can impact the geometry (i.e., shape and landward extent) of the seawater interface^61,102,128,129,138,139^.

We generate a series of randomly distributed hydraulic conductivity models with values intended to span the anticipated ranges for karstic systems at the reference site (see Fig. 5) as provided in Supplementary Information Table S7. The code to generate these distributions is provided in Supplementary Information Section 7, along with input parameters for generating these distributions.

**Figure 22:**
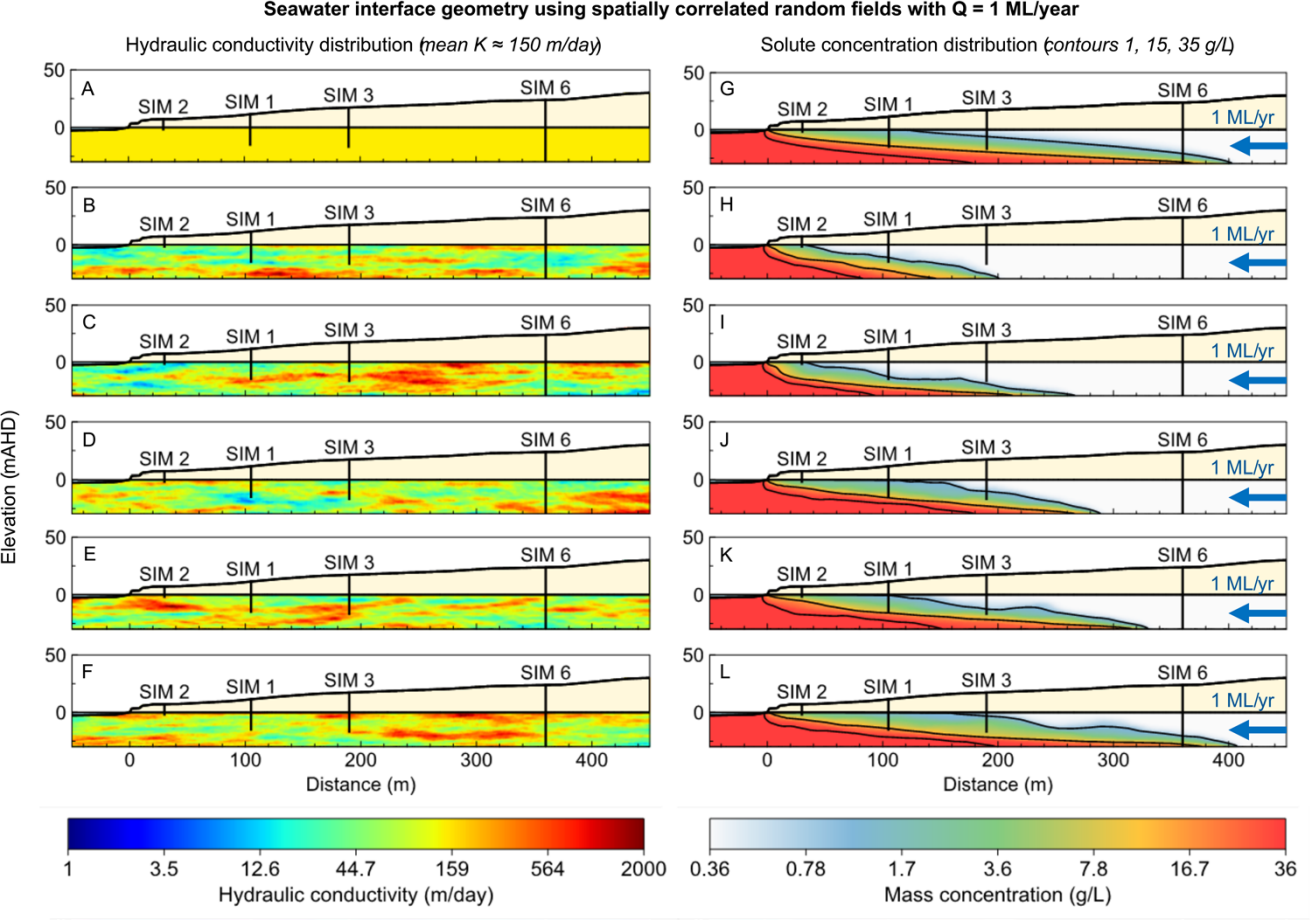
Images showing the hydraulic conductivity distribution (left column) and associated seawater interface geometry (right column) using spatially correlated random fields with a range of seeds. Despite having identical groundwater throughflow and statistically similar hydraulic conductivity distributions, the position of the seawater toe and the geometry of the wedge are significantly different.

The hydraulic conductivity distribution and associated solute concentration distribution for a subset of the models computed are shown in Fig. 22. These distributions may all plausibly exist within the Tamala limestone at the Quinns Rocks reference site. Although the groundwater throughflow is set to a constant 1 ML/year, we observe significant differences in the position of the seawater toe and the geometry of the interface. The inland position of the seawater toe ranges from 201 m (Fig. 22B) to 410 m (Fig. 22F) in the examples shown. Despite having the same groundwater throughflow and statistically similar hydraulic conductivity distribution, the distance inland from the ocean has doubled.

A key observation from Fig. 22 is that a low hydraulic conductivity zone located relatively close to the shoreline (e.g., Fig. 22B,C) will result in a steeper solute concentration gradient and decrease the landward extent of the seawater interface toe (e.g. Fig. 22H,I). In the example (Fig. 21K,L) higher hydraulic conductivity zones in the same area result in a flatter solute concentration gradient with the toe located further inland. Based on this, we suspect that the hydraulic conductivity of the near shore may be of greater consequence to the shape of the wedge than the distributions further inland. Establishing the hydraulic conductivity of this zone can be challenging due to tidal oscillations, high contrast in water density and the relatively extreme velocities of the groundwater moving into the zone of submarine groundwater discharge.

Throughout this research we have shown that measuring the water level and EC at the screened interval of several wells may be insufficient to describe the dynamic and spatially complex hydrogeology found in karstic regions. The randomly generated models shown here further highlight the requirement to acquire specific measurements at coastal margins. This raises the question: “What set of measurement *are* necessary to explain the hydraulics and solute concentration distribution for karstic aquifers?”

Geophysical methods are often used to infer the hydrogeology of shallow aquifers, particularly the seawater wedge. Electrical resistivity imaging (ERI) is a popular technique with proven application for imaging the seawater wedge^57,140–142^. However, there are aspects of application ERI at coastal margins that need investigation. We demonstrate and discuss these in the next section.

## Part 5. Geophysics: How Reliable is Electrical Resistivity-Based Monitoring?

Conventional groundwater monitoring with wells can provide high-quality local information, but as we have shown, may be insufficient to progress to a robust solute transport model. Geophysical measurements provide a method to investigate the volume of earth between wells; however, the imaging outcomes are subject to a range of interpretations and uncertainties. We will demonstrate a process by which solute concentration distribution from numerical groundwater modelling can be used to create numerical simulations of ERI outcomes, which can then be compared to field data.

A desirable outcome from electrical resistivity imaging (ERI) is that it recovers an accurate representation of the shallow subsurface resistivity. ERI field measurements are acquired by passing current across a pair of electrodes, while measuring the voltage drop across a second pair of electrodes. The number, orientation, spacing, and geometry of the two electrode pairs (i.e. the quadrupoles) relative to the earth’s electrical structure will impact the methods ability to construct a reasonable subsurface image of electrical conductivity by inversion^143^. The dipole-dipole configuration, or combinations of dipole-dipole with other electrode arrays are considered highly reasonable for recovery the hydrogeology at the seawater interface^57,144^.

### Converting solute to formation resistivity

The relationship between total dissolved solids in solution (TDS) and water resistivity is typically near-linear^99,145^. If water resistivity is known it can then be converted to an estimate of formation resistivity via Archie’s law^146,147^.

5 $$\begin{array}{c}{\rho }_{b}=a{\rho }_{w}{\phi }^{-m}{S}^{-n}\end{array}$$

where ${\rho }_{b}$ is the formation electrical resistivity (Ω m), ${\rho }_{w}$ is the fluid electrical resistivity (Ω m), and $\phi$ is the porosity. The tortuosity factor$\,a$, and cementation exponent$\,m$, can be empirically related to the formation rock type^146,148^. *S* is the fluid saturation (between 0 and 1) and *n* is the fluid saturation exponent.

The formation resistivity is dependent on the choice of *a*, and *m*. Porosity in the Tamala Limestone is highly variable, from 0.2 to 0.5^31^. Tortuosity and cementation factors are based on generalised values from literature^149,150^. Table 3 details suggested ranges of values and provides an example of the formation resistivity for a given groundwater solute concentration. For the parameters we choose, fresh groundwater and highly saline groundwater are expected to measure ~198 Ω m and 2.8 Ω m respectively.

**Table 3:** Archie’s law ^146^ parameters showing variation in formation conductivity from values of ‘a’ and ‘m’.

Parameters	Literature Values^31,149,150^		
	Sandstone	Carbonate	Simulated Values
Porosity ($\phi$)	—	0.2–0.5	0.3
Tortuosity Factor ($a$)	0.515–3.45	0.425–1.51	1
Cementation Factor ($m$)	1.225–2.20	1.685–4.14	2.2
Saturation ($S$)	—	—	1
Saturation Exponent ($n$)	—	—	2

Archie’s Law was originally intended for clean sandstones^149^; however there are many variations of the law including shaly sands^151,152^, multiple liquid phases^153^, and other various modifications^147,154,155^. The values in Table 3 below are reasonable in the context of observations from well logging and ERI experiments completed in the Tamala limestone^31,32,91^.

In some circumstances, formation resistivity can be derived from hydraulic conductivity^156–158^; however, such relationships must be applied with considerable caution. The potential for error exists where fluid salinity is not constant, or where clays are present. Beaujean, *et al*.^138^ use a modified version of Archie’s law to include a relationship between hydraulic conductivity and the bulk electrical conductivity.

In coastal settings like Quinns Rocks it is highly likely that the distribution of saline and fresh water is the primary driver for the large-scale formation resistivity distribution as would be measured by an ERI survey.

### Model generation and inversion

ERI data consists of geometry (i.e. locations of electrodes), transmitted current, and measured voltage. Inverse modelling with this data is completed to recover the subsurface distribution of electrical resistivity (i.e. the formation resistivity). The forward ERI problem is defined by the distribution of electrical fields in the ground due to a point current source. This equation is^159,160^

6 $$\begin{array}{c}-\nabla \cdot [\sigma (x,y,z)\,\nabla \phi ({\rm{x}},{\rm{y}},{\rm{z}})]=I\delta (x-{x}_{s})\,\delta (y-{y}_{s})\,\delta (z-{z}_{s})\end{array}$$

where $\sigma$ is conductivity of the medium (S/m), $\phi$ is electric field intensity (V/m), $I$ is the current source (A), $\delta$ is the Dirac delta function. Inverse modelling (i.e. inversion) of geo-electrical measurements then creates a resistivity model that generates the same voltages as a set of field observations.

Electrical resistivity datasets were simulated for a formation resistivity distribution derived from solute concentration distribution using a framework built on pyGIMLi^161^. We add 5% voltage dependent noise plus 1 $\mu V$ absolute error as recommended by Friedel^162^. All scripts are freely available from the authors for review and re-use.

The synthetic data is inverted using RES2DINV^163^, a commercially available resistivity and IP inversion software. It uses a modified smoothness constrained least-squares inversion method with variable smoothness and roughness constraints^163,164^. Inversion of geo-electrical data requires selection of many parameters that can affect electrical resistivity imaging outcomes. Key parameters include the regularisation (i.e. damping factor), error norm^57,165–167^, topographical relief, a-priori inputs, and the acquisition array^143,168–170^

Our numerical modelling uses a hybrid dipole-dipole and Schlumberger electrode configuration identical to that used to acquire field data at the Quinns Rocks reference site in 2015^57^. The survey used 44 electrodes spaced at 10-metre increments. The survey extends from the shoreline to residences located 430 metres inland. Further analysis of the field data can be found in Costall, *et al*.^57^. All inversions are completed with a finely discretised quadrilateral mesh with robust inversion constraints^171^. Our data is freely available, and outcomes can be replicated using the inversion parameters supplied in Supplementary Table S8.

**Figure 23:**
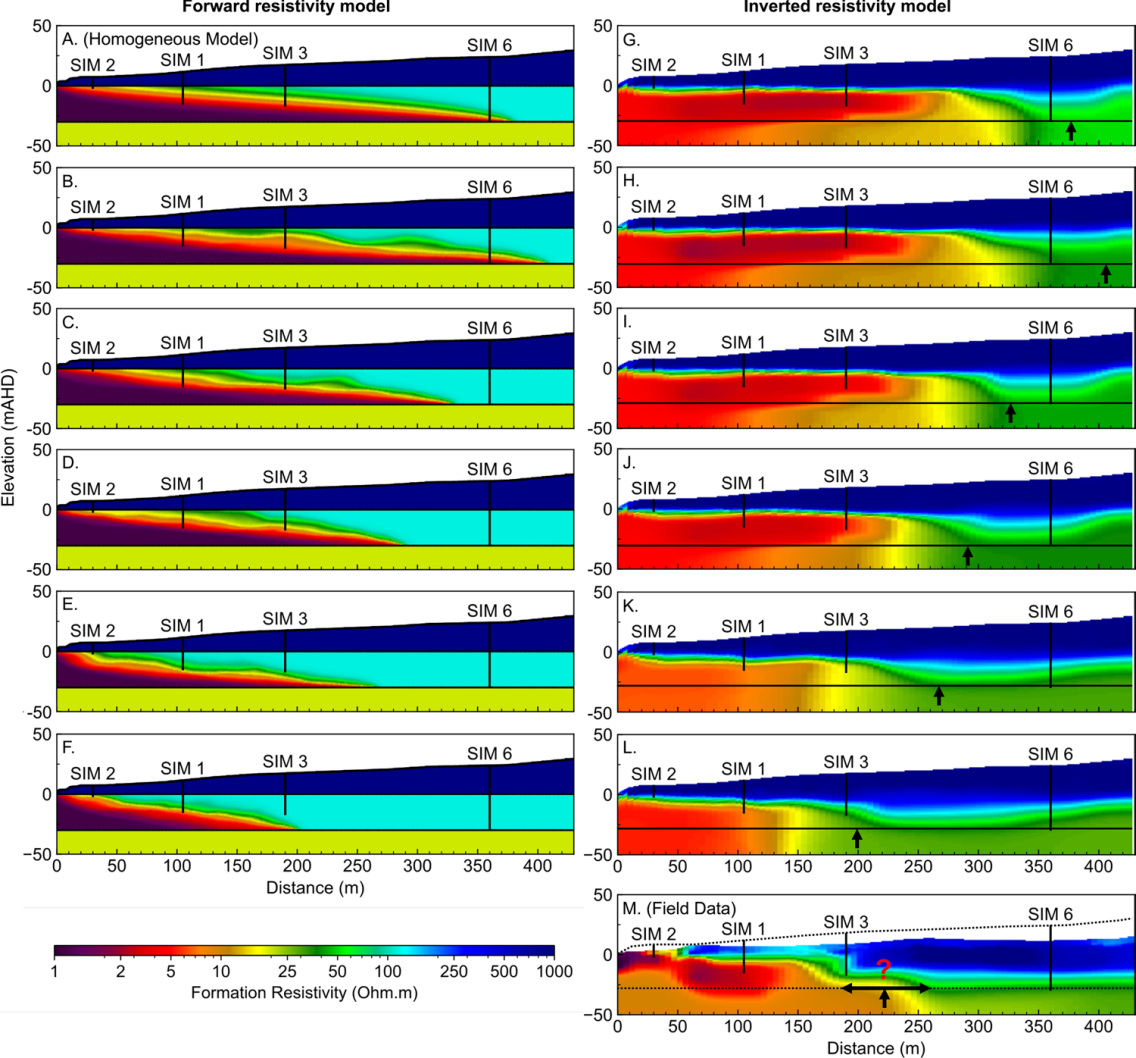
Images showing the inverted resistivity distributions compared to the forward resistivity distributions for a range of hydraulic conductivity models. Panel A shows the forward resistivity distribution for a homogeneous model with 150 m/day hydraulic conductivity (e.g. Fig. 22G). Panels B through F show the forward resistivity model based on the solute distribution for each of the randomly generated hydraulic conductivity distributions (e.g. Fig. 22H through L). Panels G through L show the inverted resistivity outcomes of the synthetic models. Panel M shows the inverted resistivity distribution from field ERI data near to the Quinns Rocks reference, including the approximate projected locations of the SIM wells and topography (dashed line). The inversion outcomes from the synthetic data show the approximate location of the seawater toe and the depth of the substrate can be recovered. However, definition of the mixing zone and the wedge geometry is ambiguous and could be easily misinterpreted.

Figure 23 shows the inverted formation resistivity distribution from models created using the solute concentration of a homogeneous aquifer model (e.g. Fig. 22A), along with the set of randomly-generated heterogeneous hydraulic conductivity models shown in Fig. 22B to F. Figure 23M shows the inverted formation resistivity distribution for the field data collected at the Quinns Rocks reference site.

A comparison of the forward models of formation resistivity derived for transport modelling and the inverted ERI resistivity distributions is shown in Fig. 23. A basic observation is that the unconstrained ERI inversion outcomes do not precisely map the distribution of resistivity within the mixing zone. The geometry of the wedge is also strongly smeared. Although the toe of the seawater wedge can be approximately located from the resistivity sections, we suspect that without the forward model present the toe could be interpreted to be well over 100 m further towards the ocean.

Note that measurements in the SIM wells show that seawater intrusion has passed SIM 3, but not SIM 6, at the time of the ERI survey. However, the resistivity imaging in Fig. 23M presents a situation where the toe could be interpreted anywhere between 180 and 270 m from the shoreline.

We have shown that using electrical imaging generates a potentially misleading reconstruction of the seawater wedge. The combination of (i) a dipping, smoothly transitioning mixing zone, and (ii) a sharp, near-horizontal high-contrast boundaries of the water table and substrate can be difficult to recover using unconstrained inversion^57,144^. The technique may be improved by using focused and optimised arrays^172,173^, novel acquisition techniques such as borehole-based imaging^57,174^, and constrained inversions^175–177^. We focus on unconstrained inversion as under most circumstances the constrains or infrastructure required for more complex imaging will not be available.

We are presented with several conclusions regarding unconstrained inversion ERI to recover the seawater interface. These are:

(i) ERI provides a valuable but highly smeared and damped version of formation resistivity distribution.
(ii) ERI cannot recover precise values of resistivity for the substrate or seawater-saturated formation.
(iii) Referencing the inverted ERI field data outcomes to sets of images derived from numerical transport modelling should be completed as a minimum standard of investigation.
(iv) ERI should not be expected to provide exact point or geometric constraints such as the location of the toe of the seawater interface.

## Part 6. Discussion: Capturing the Dynamic Hydrogeology of Coastal Margins

For any experiment, sufficient temporal and spatial sampling with reference to the desired outcome is fundamental. This also applies to field scale measurement systems deployed for hydrogeology. The hydraulics at coastal margins has significant differences compared to most hydrogeological settings. In Part 6, we will compute the distribution of Darcy flow velocity across the seawater interface and will later consider implications for the type of monitoring systems that may be suitable for characterising this highly dynamic setting. Also, we suspect that methods for measuring groundwater flow velocity itself may present useful and currently under-utilised pathways to characterising shallow high-quality coastal aquifers for both research and aquifer management purposes.

### Can we use groundwater velocity to measure seawater intrusion?

There are extreme changes in flow velocity associated with the interface between seawater and fresh groundwater. Fresh terrestrial groundwater drives towards the coast but cannot pass through the denser seawater. The Darcy velocity of the fresh groundwater must increase as the flow area decreases towards the ocean^178^.

First, we illustrate these extremes in velocity contrast for a homogeneous aquifer. We then compare these results with flow regimes for an aquifer with a complex hydraulic conductivity distribution, as might be expected in karstic coastal aquifers. The homogeneous aquifer model it taken from Fig. 17 (i.e. 200 m/day hydraulic conductivity with a 30 m saturated thickness), except with a greater groundwater throughflow of 5 ML/year.

**Figure 24:**
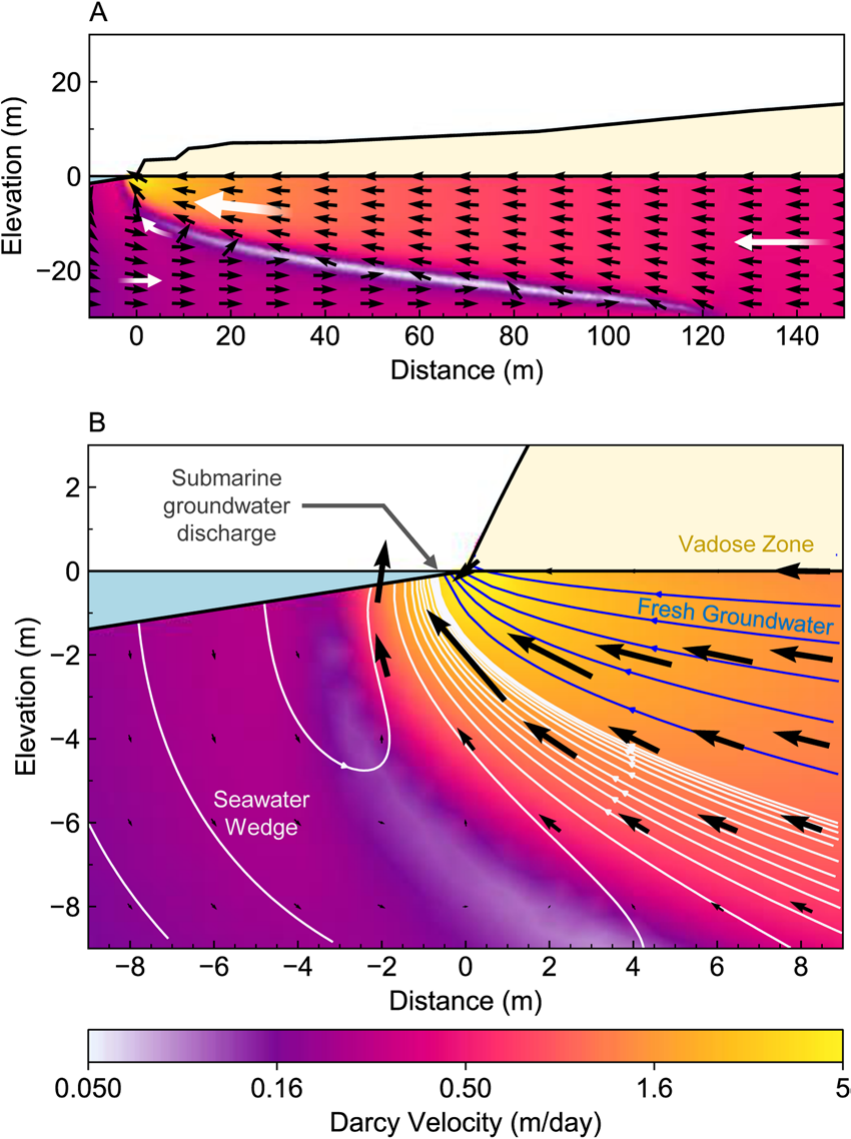
Images highlighting the extremes in Darcy velocity (m/day) that occur across a seawater wedge for a homogeneous aquifer. The velocity of fresh groundwater flowing towards the coast increases as the available area of flow decreases above the dense seawater wedge, until the fresh groundwater exits the system through the zone of submarine groundwater discharge (highlighted in Panel B). The velocity of groundwater proximal to the submarine discharge is an order of magnitude greater than the velocity of the fresh groundwater prior to being restricted by the seawater wedge. In contrast, the groundwater velocity in the seawater recirculation cell can flow in the opposite direction (i.e. towards the land) and is significantly less than that for the fresh groundwater above.

Three distinct Darcy velocity regimes can be identified from Fig. 24. These are; (i) groundwater with negligible velocity (10^−9^ m/day) at the seawater wedge face, (ii) groundwater with high velocity (~5 m/day) discharging to the ocean and (iii) groundwater with uniform velocity passing through the aquifer on the landward side of the seawater wedge (~0.4 m/day). The three flow regimes are spatially related to the seawater interface.

Monitoring wells located near to the seawater interface could observe several orders of magnitude variation in groundwater velocity, if the seawater interface moves a relatively small distance across the screens. These significant changes suggest that new seawater intrusion monitoring systems able to capture detailed vertical and horizontal changes in groundwater flow velocity and solute concentration distribution may be necessary to understand groundwater behaviour along coastal margins.

**Figure 25:**
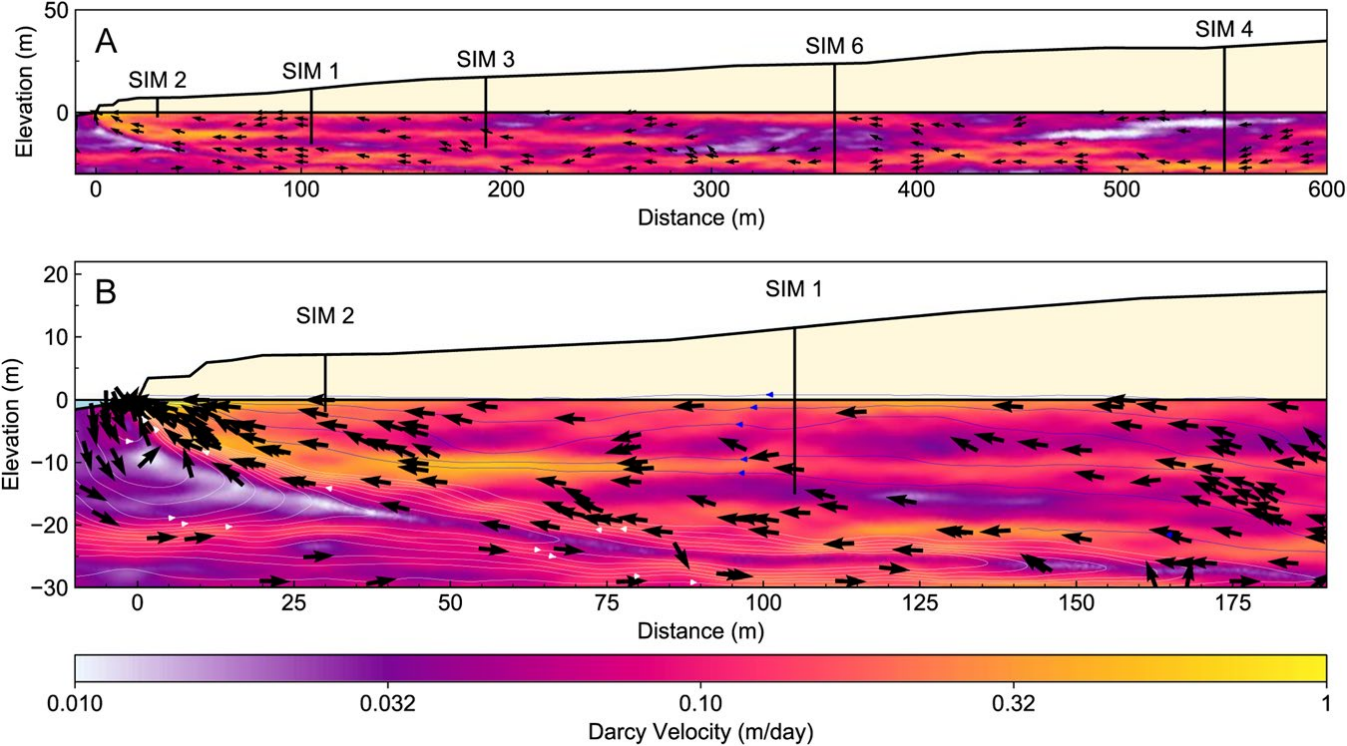
Images showing the Darcy velocity distribution for a complex hydraulic conductivity model (see Fig. 22F). The distribution of arrows is weighted by the magnitude of the y-component. Panel A shows the full width of the model including zones of relatively high and low velocities related to the hydraulic conductivity distribution. Panel B show a zoomed in section of Darcy velocity proximal to the seawater wedge. It highlights the extremely slow groundwater velocity in the seawater recirculation cell and the complex flow directions related to hydraulic conductivity distribution in the zone of fresh terrestrial groundwater above the wedge. The screened intervals in the monitoring wells may be in high-velocity conduits or low-velocity pockets that may result in higher or lower inter-well hydraulic conductivity gradients. A distributed velocity measurement along the length of the well may identify these zones and improve calibration needed for solute transport modelling along coastal margins.

We extend analysis of Darcy velocity to the heterogeneous random-field examples first shown in Part 4. The distribution of arrows in Fig. 25 is weighted by the y-component of the directional vector, highlighting areas with non-horizontal flow. A zone of extremely low velocity exists along the seawater/freshwater interface. This was also seen in the homogeneous model (Fig. 24), however in Fig. 25 we can contrast flow along hydraulically connected pathways with zones of low hydraulic connectivity that strongly affect flow paths and pressure gradients within the aquifer.

From this flow distribution, we conclude that the precise location of screened intervals for wells within a seawater intrusion monitoring network will likely affect the interpretation of hydraulic parameters. If we consider a pair of well screens set in a hydraulically connected conduit, the water level gradient between these wells would appear to be exceedingly low. However, these gradients will not reflect the hydraulics of full aquifer system and could lead to a strongly biased interpretation or difficulties in calibration from numerical groundwater modelling.

Our analysis suggests that additional information concerning vertical distribution of hydraulic properties, such as aquifer pressure or flow velocity distribution could significantly improve hydrogeological characterisation of near shore settings. Observations from Figs. 23 and 24 provide key insights required for design of new distributed seawater monitoring systems. They also link back to the difficulties encountered when attempting to interpret gradients between wells that may have screens set in rocks with significant difference hydraulic conductivity as observed throughout Part 3. The analysis of field data combined with numerical modelling and the visualisation of Darcy velocity for complex hydraulic conductivity distributions (i.e. as found at reference site) points to the need for new monitoring strategies. Future options (Fig. 25 and Table 4) for seawater intrusion monitoring will be presented in the conclusions.

**Table 4:** Options for instrumentation and measurement of seawater intrusion monitoring wells (see Fig. 26).

	Solution (see Fig. 26)	Advantages	Disadvantages	Examples
Pressure Distribution	Vertically distributed pressure sensors: Permanent monitoring outside of casing allows for measurements of true point pressures in depth.	Direct measurement of pressure without potential error associated with hydraulic head. Low per-sensor cost and automated measurement.	Difficult or impossible to recover sensors if cemented to outside of casing and can increase the well establishment costs.	Smerdon, *et al*.^179^ Strout and Tjelta^180^
	Spatial distribution of monitoring wells: Measure the variation of hydraulic properties along and away from the shoreline.	3D characterisation of aquifer properties and variation parallel and perpendicular to the shoreline.	Greater number of wells required for high quality characterisation.	Coscia, *et al*.^181^
Groundwater Velocity	Flowmeters: Direct measure of flow velocity at multiple points within the aquifer.	Direct throughflow measurement that removes the uncertainty of gradient-based estimates.	Groundwater velocity can be highly variable in karstic environments, requiring multiple measurements.	This work (Part 5) Bayless, *et al*.^189^
	Tracers: Groundwater flow estimation and hydraulic parameterisation.	High quality estimates of various hydraulic parameters, such as dispersivity, and flow velocity.	Limited selection of tracers compatible with seawater chemistry. May require repeated physical water sampling.	Jørgensen, *et al*.^190^ Stuyfzand^191^ Pulido-Leboeuf, *et al*.^192^
Geology (Dip, facies changes, etc.)	Surface/Borehole GPR	High-resolution imaging of dipping layers and geology	Requires a high electrical resistivity environment for reasonable depth-of-investigation (e.g. non-saline water).	This work (Part 2)
	Wireline logging data (Nuclear Magnetic Resonance, neutron logs, full waveform sonic)	High-quality wireline information along extent of borehole (e.g. can recover bound, free and capillary water porosity along with permeability estimates)	Cost of establishment, instrumentation and repeat logging. Measurements require repeat access to open borehole (i.e. without instrumentation) and are manual logged.	Smith, *et al*.^31^ Almalki, *et al*.^193^ Mathieu and Toksö: z^194^
	Distributed Acoustic Sensing (DAS)	High-resolution seismic imaging capable of recovering local hydrogeology. Readily automated.	Fibre-optic technology still developing and will need specifically engineered optical fibre for sufficient near-surface resolution. Interrogators are expensive.	Schenato^182^ Shanafield, *et al*.^183^ Parker, *et al*.^184^
	Distributed Temperature Sensing (DTS)	Low-cost, low maintenance, thermal transport measurements, shared system with DAS. High accuracy temperatures.	Limited spatial resolution of thermal property.	Selker, *et al*.^185^ Bense, *et al*.^186^ Shanafield, *et al*.^183^
	High frequency ‘sparker’ seismic source	High-frequency source (plus 1000 Hz) for the high-resolution seismic imaging (combined with DAS system).	Requires access to borehole, and difficult to automate if required.	Rechtien, *et al*.^195^
	Petrophysical analysis of core samples	High-value constraints on physical and hydraulic parameters (porosity, anisotropy, dip angle, permeability).	High cost of recovering samples, which can be damaged during recovery. Further testing of samples is required. Information is highly localised.	Maliva^196^
Solute Concentration Distribution	Water electrical conductivity sensor arrays (Galvanic, inductive, fibre-optic measurements)	Direct conductivity measurement, used with ERI for defining the formation factor.	Longevity of the electrodes (conductive probe vs inductive probe).	Possetti, *et al*.^197^ Hilhorst^198^ Esteban, *et al*.^199^
	Cross-well electrical resistivity imaging	High-resolution electrical resistivity imaging and time-lapse imaging of electrical conductivity.	Limited by well location, and requires dedicated engineering at time of well development	Ogilvy, *et al*.^200^ Turner and Acworth^201^
	Surface-based electrical resistivity imaging	Flexible acquisition geometry (i.e. not limited to wells), and time-lapse imaging.	Lower resolution than cross-hole, with loss of resolution with depth. Deployment and automation may be difficult in urbanised areas.	This work (Sect. 4)
	Multi-electrode borehole monitoring	*In-situ* time-lapse measurement of electrical resistivity with localised imaging capability.	Non-flexible acquisition geometry and requires specific engineering at time of well development.	Grinat, *et al*.^202^ Grinat, *et al*.^203^
	Time lapse induction logging	High-resolution formation conductivity proximal to well.	Difficult to automate and requires specific well construction (PVC/FRP). Other *in-situ* instruments may affect measurements.	Spies^204^
	Water chemistry analysis	Repeatable and low-impact water chemistry sampling from a known depth.	Requires dedicated equipment established during well construction.	Freifeld, *et al*.^205^

Our research identifies a range of challenges in recovering hydraulic properties and solute concentration distribution at *the seawater interface. These challenges can be resolved by improved monitoring technologies. The monitoring solutions suggested below* are applicable to both industry and research objectives. They should be tailored for site-specific conditions and objectives. Although the initial cost may be higher for automatic real-time monitoring of near-shore groundwater systems, the long-term costs may be lower (e.g. by replacing manual monitoring) and benefits from such systems will likely significantly outweigh initial expenditure.

## Conclusion

The interplay between vast quantities of terrestrial groundwater discharging into the ocean and the landward ingress of seawater is central to the health of coastal aquifer systems. People and environments on all continents interact with, influence, and rely on these systems daily. The loss of high-quality coastal groundwater to seawater intrusion is a serious concern globally.

The relationship between groundwater throughflow and seawater intrusion has been dissected with numerical simulation and field data from an established seawater monitoring site in the south west of Western Australia. The site has over 30 years of conventional well-based seawater monitoring data. Here seawater intrusion has paralleled declining rainfall, rapid increase in population density, development of a regional water supply, and rising sea levels. We find that decoupling these influences is impossible without specific information concerning hydraulics and groundwater chemistry. We show that, even with the abundance of data collected at the reference site, the rate of intrusion and geometry of the interface could not be determined with confidence.

Locating the landward extent of the seawater interface is important and challenging in highly heterogeneous karstic aquifers. We found few studies that integrate long-term seawater intrusion monitoring field data into critical analysis of data acquisition strategies and various modelling approaches. We demonstrate that significant error can result from using simple analytical solutions to determine the landward extent of the seawater interface.

The landward extent of the seawater wedge is often used to guide coastal groundwater management decisions. However unresolved subsurface hydraulics may lead to considerable error in these estimates. Ground Penetrating Radar is used to reveal anisotropy and heterogeneity in karstic groundwater settings. We demonstrate the impact of dipping hydraulic anisotropy on the landward extent of seawater. Sets of images show how increasing the hydraulic anisotropy rotation angle can shift the seawater interface significantly closer to the ocean. Alternatively, the rotation angle of hydraulic anisotropy can be manipulated to maintain the landward extent of saline groundwater for vastly different aquifer throughflows.

Heterogeneity in hydraulic conductivity can also strongly impact the landward extent of the seawater interface. The outcomes from our numerical experiments with plausible spatially correlated random fields highlight the range of seawater interface geometries that can occur for systems with the same average hydraulic conductivity and groundwater throughflow. A key finding from these experiments is that the hydraulic conductivity of the aquifer proximal to the zone of submarine groundwater discharge is a key driver for the geometry and landward extent of the seawater wedge. The level of detail required to numerically simulate these heterogeneous environments cannot be obtained from conventional monitoring data which recovers water level and EC measurements from one depth interval.

Surface-based electrical resistivity imaging (ERI) is a valuable tool to establish the geometry of the seawater interface; however, determining the precise location of the toe requires the combination of ERI and solute transport modelling. That is, transport modelling is used to simulate the seawater wedge, then the solute concentration distribution is converted to formation conductivity and synthetic ERI data sets created for different wedge geometries. The simulated ERI data is inverted with the same parameters used for the field ERI data. Finally, imaging from synthetic and field data are systematically compared. Used appropriately, ERI can partially mitigate the problem of under-sampling from conventional sparsely distributed monitoring wells but – as we demonstrate through modelling of heterogeneous aquifers – it cannot mitigate the uncertainty in measuring or predicting groundwater throughflow.

**Figure 26:**
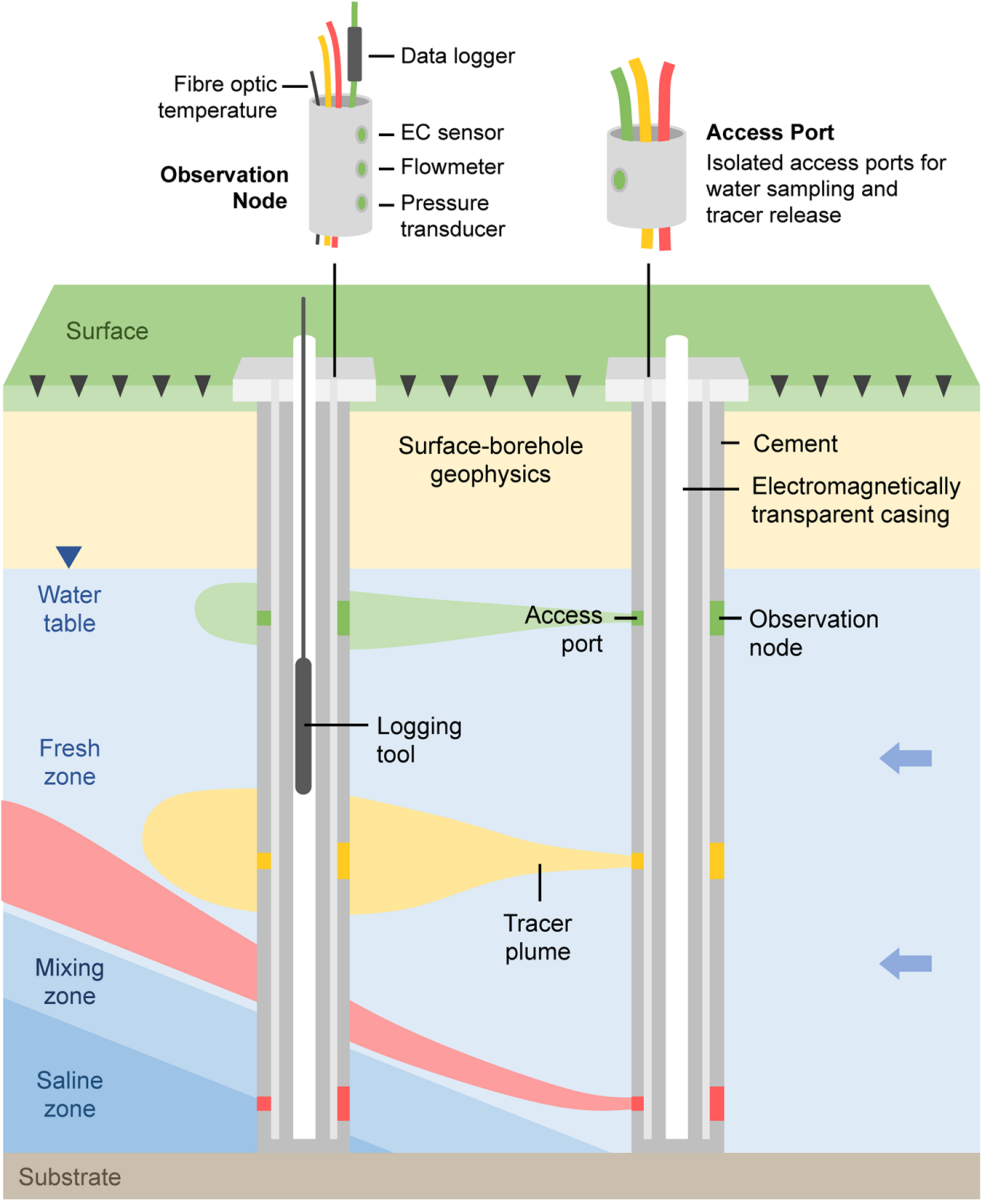
Schematic of a seawater intrusion monitoring system that would provide sufficient evidence to characterise the seawater interface in a shallow coastal aquifer. The system has multiple sampling nodes at various depths within the aquifer, with a suite of *in-situ* monitoring devices. These include *in-situ* flow meters, EC meters, and pressure piezometers. The data collected by this setup is well suited to reveal the subtle dynamics of a coastal aquifer system.

From our analysis of well-based monitoring data, numerical groundwater modelling, and representations of the extremes in groundwater velocity, we find that subsurface monitoring technologies need to be specifically designed for the nearshore coastal settings. Options for monitoring the dynamic relationship between solute concentration distribution, hydraulic property distribution, and groundwater throughflow at coastal margins are presented in Fig. 26 and Table 4.

The schematic in Fig. 26 proposes an arrangement of monitoring devices for a multi-well system capable of automated, real-time, and highly discretised sampling of hydraulic parameters. Table 4 details the seawater intrusion monitoring technologies that may be deployed within such a system of wells. These are provided under four categories including: (i) measurement of pressure distribution, (ii) subsurface flow characterisation, (iii) characterisation of geology (including hydraulic parameter distribution), and (iv) recovery of groundwater chemistry distribution.

These options can provide quantitative information required to understand and simulate shallow karstic hydrogeological systems along coastal margins. For example; the automated measurement of the vertical distribution of EC, flow velocity, and pressure data^179–181^, recovery of groundwater samples at specific depths for periodic chemical analysis or tracer release, and a distributed fibre optic system capable of measuring temperature and strain distribution (i.e. via engineered fibre) can provide highly discretised measurements throughout the aquifer^182–186^.

There is clear evidence for seawater intrusion and increased stress on coastal groundwater systems worldwide. We hope the insights and practical conclusions from this research will lead to a new era where automated real-time groundwater monitoring systems along coastal margins appropriately inform predictive groundwater modelling that ultimately leads to better management of coastal aquifers systems.

## Supplementary information

Supplementary Information.
