# Publisher Correction: Groundwater Throughflow and Seawater Intrusion in High Quality Coastal Aquifers

**DOI:** 10.1038/s41598-020-75736-9

**Published:** 2020-10-30

**Authors:** A. R. Costall, B. D. Harris, B. Teo, R. Schaa, F. M. Wagner, J. P. Pigois

**Affiliations:** 1 Western Australian School of Mines: Minerals, Energy and Chemical Engineering, Curtin University, Curtin, WA Australia; 2 Institute for Applied Geophysics and Geothermal Energy, RWTH Aachen University, Aachen, Germany; 3 Department of Water and Environmental Regulation (DWER), Joondalup, WA Australia

Correction to: *Scientific Reports* 10.1038/s41598-020-66516-6, published online 17 June 2020

This Article contains an error in Figure 17, where the graph data in panels (D) and (E) are a duplication of panel (C). The correct Figure 17 appears below as Figure 1.

**Figure 1:**
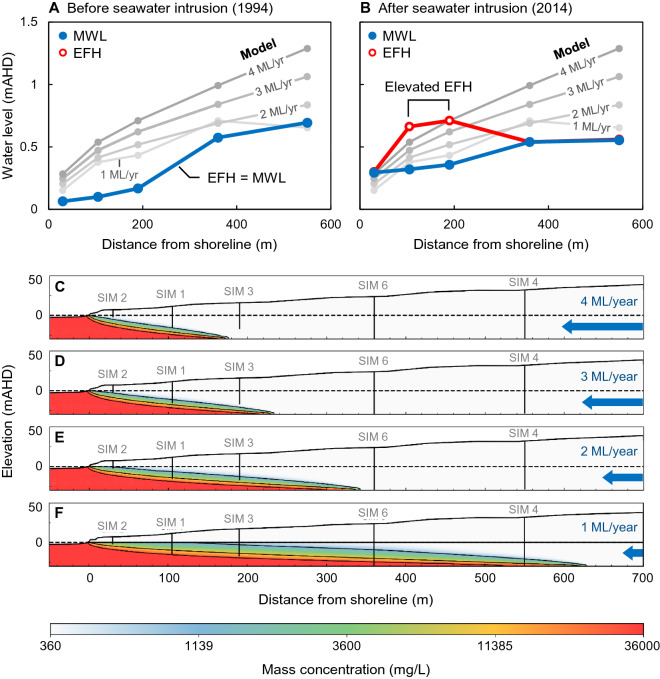
Set of images showing the simulated seawater interfaces for a range of groundwater throughflow in a 30 m thick aquifer with hydraulic conductivity 200 m/day over an impermeable substrate (i.e., average values for the Quinns Rocks reference site). Charts A and B show the measured water levels (MWL) and equivalent freshwater head (EFH) in 1994 and 2014. The EFH is calculated assuming that the groundwater at the well screen occupies the entire well column. Images C, D, E and F show the solute concentration distribution corresponding to groundwater throughflow of 4, 3, 2, and 1 ML/year respectively. According to this homogeneous aquifer model, groundwater throughflow must remain above 2 ML/year at Quinns Rocks to maintain fresh groundwater at SIM 6; however, this results in significantly greater simulated hydraulic head than the field observations. We find that there is no combination of hydraulic conductivity and throughflow for a homogeneous aquifer that can reasonably explain both measured values of hydraulic head and solute concentration at the reference site. This points towards high contrast in hydraulic parameters within the aquifer as a strong influence on the landward extent of saline groundwater.

Additionally, in Figure 20, where the graph data in panels (B) and (D) are a duplication of panels (A) and (C). The correct Figure 20 appears below as Figure 2.

**Figure 2:**
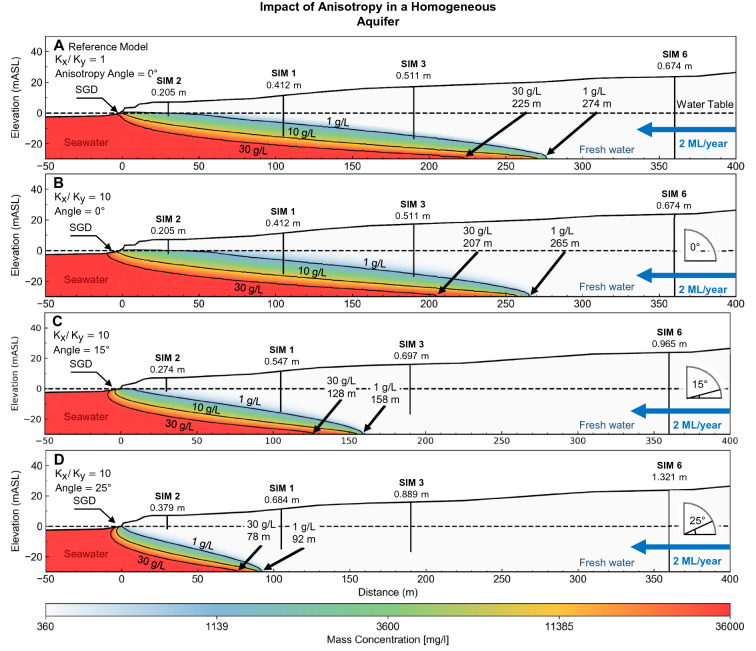
Images showing the influence of increasing anisotropic angle on the seawater wedge geometry in a high hydraulic conductivity (200 m/day) homogeneous aquifer. In this example, the angles of anisotropy are 0° (**B**), 15° (**C**), and 25° (**D**) degrees for a constant anisotropic ratio *Kx/Ky* = 10. Panels A and B compare the isotropic and anisotropic models. The increasing angle of anisotropy is associated with higher hydraulic heads (annotated below each well), which is likely to be the primary driver behind the seaward movement of the seawater interface. The seawater wedge geometry in Panel D resembles the seawater interface geometry for a homogeneous isotropic model with a groundwater throughflow rate of 4 ML/year (see Fig. 15). This demonstrates that knowing the position of the wedge toe is not a reliable indicator of throughflow and vice versa.

Lastly, in Figure 21, where the graph data in panels (A), (C), and (D) are a duplication of panel (B). The correct Figure 21 appears below as Figure 3.

**Figure 3:**
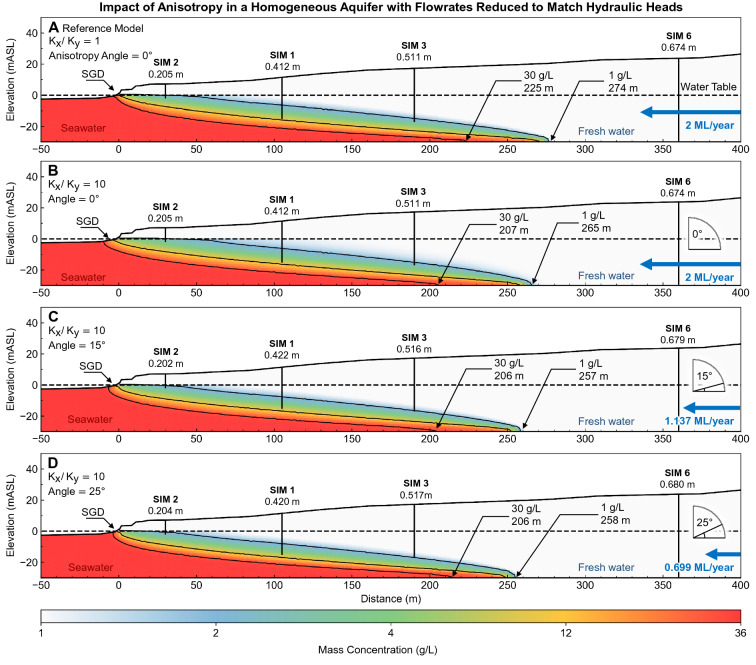
Images showing the influence of anisotropy on the seawater wedge geometry after reducing groundwater throughflow to match hydraulic heads. The differences between the resulting seawater wedge geometry is minor. For example, the wedge geometry from the lowest flowrate (0.69 ML/year) with anisotropic angle of 25° (Panel D) is similar to the wedge geometry at high 2 ML/year with an angle of 0° (Panel A). Lower anisotropic angles result in a wider zone of submarine groundwater discharge. This figure highlights the fact that knowing the seawater wedge position alone is not an indicator for groundwater throughflow. There is a clear need for better constraints on hydraulic parameters to understand the seawater interface in these coastal aquifers.

