# Supplementary material for: Groundwater Throughflow and Seawater Intrusion in High Quality Coastal Aquifers
Source: Sci Rep. 2020 Jun 17;10:9866. doi: 10.1038/s41598-020-66516-6 (PMC7300005; doi:10.1038/s41598-020-66516-6)
Supplement: Supplementary file 1 — Supplementary Information. [file 41598_2020_66516_MOESM1_ESM.docx]

Supplementary Information

**Title**: Groundwater Throughflow and Seawater Intrusion in High Quality Coastal Aquifers

**Authors**: A. R. Costall¹*, B. D. Harris¹, B. Teo¹, R. Schaa¹, F. M. Wagner², J.P. Pigois³

This document contains information that is supplementary in nature to the main research document. Examples include processing flows, comparative tests, and tables of solutions that are not explicitly required in the manuscript but may be of interest to researchers or required to reproduce results.

All scripts, data, models, and any other information, can be found online or on request from the authors.

# Table of Influential Parameters on Seawater Interface Model

Supplementary Table S1 lists some of the factors that can influence simulation outcomes of a seawater interface in karstic limestone aquifers. The table is split into temporal (i.e. time-varying) and spatial (varies with distance/depth) factors. Many of these factors are difficult to predict and may result in over-complication of models with regards to intended outcomes.

Supplementary Table S1: Temporal and material variables that can influence the geometry of the seawater interface.

|  | **Factor** | **Comment** |
| --- | --- | --- |
| Temporal | Climate change (sea-level rise) | Global sea-level rise can result in the seawater wedge intruding further inland. The impact of sea-level rise on the seawater interface is a popular research topic and there are many examples of increased seawater intrusion due to global sea level rise ^1-6^. |
|  | Tidal forcing and wave-surge | Daily tidal oscillations causes the very shallow (< 2 m) near-shore salinity contours to vary due to infiltration of seawater in the upper-beach face ^7^. This can potentially double the width of the mixing zone without significant changes in the position of the seawater toe ^8^. |
|  | Seasonal variation in recharge | Seasonal variation in rainfall recharge results in a dynamic groundwater throughflow, which will affect the seawater interface. Other periodic cycles in ocean and groundwater levels, such as the Spring/Neap and semi-diurnal cycles also influence the seawater mixing zone and seawater circulation cell ^9,10^. |
| Material | Caves and conduits | High-permeability karstic aquifer systems often contain networks of extremely high permeability caves and conduits. The orientation and density of conduits affect the geometry of the seawater interface ^11,12^. |
|  | Substrate Angle | Seawater intrusion models typically assume the lower confining substrate is both flat and impermeable, however the geometry of the seawater interface is sensitive to the angle of the substrate, such that an angle of 1% (i.e. 1 m over 100 m) bed slope can move the seawater toe approximately 30–50% further inland ^3^. |
|  | Dispersivity | Dispersivity is often highly variable and site-specific, with a range of values used throughout literature ^13^. For example, in a one kilometre-long unconsolidated aquifer, dispersivity estimates can range from 1.31, to 132 ^14^. Lowering the dispersion coefficients will reduce the zone of dispersion along the seawater/freshwater interface and tends to increase the landward extent of the toe ^15,16^. |
|  | Ocean Interface Slope & Bathymetry | The shape of the intrusion face affects the geometry of the seawater interface. Examples of the marine-side face of seawater intrusion models can range from bathymetric representations (e.g. this research), to simplified wedges, and even vertical intrusion faces. High gradient slopes at the seaward wedge may reduce the simulated length of seawater intrusion, increase the residence time of seawater in the wedge, and reduce the zone of submarine groundwater discharge ^17,18^ |
|  | 3D Geometries | Cave systems and conduits exist in a 3D space, and groundwater will flow preferentially through these pathways. A 2D groundwater model may not encapsulate the complexity of the seawater wedge in these environments ^19^. Expansion to a 3D simulation space exacerbates many of the uncertainties identified throughout this paper without guarantee of more-realistic simulation outcomes. |

# Model Details and hydraulic parameters local to the Quinns Rocks reference site

Hydraulic parameters and the groundwater allocation limits for the superficial aquifer north of Perth are provided in Kretschmer and Degens ^20^. Hydraulic conductivity estimates range from 7 to 427 m/day. Nidagal and Davidson ^21^ use a hydraulic conductivity of 200 m/day in estimations of groundwater throughflow. Kretschmer and Degens ^20^ suggest a lower value of 130 m/day is appropriate, based on the mean value from local tidal time lag aquifer tests.

Recharge estimates cover the flow net cell ‘N’ ^20^. The dimensions of the cell are 2040 wide (perpendicular from the coast), 1080 m long (parallel to the coast), with an estimated aquifer thickness of 30 meters. Volume-based calculations (e.g. fluid-flow) are calculated using an aquifer width ($\Delta x$) of one unit-length, (e.g. 1-meter) ^22^. The average Darcy flux through the throughflow surface of the model per unit area is therefore:

$$\begin{aligned} \bar{v}=\frac{\bar{Q}}{A}=\frac{\bar{Q}}{\Delta x\Delta z} \#\left( 1 \right) \end{aligned}$$

$\begin{aligned} \bar{v}=\frac{3484 ML\text{/year}}{1080 m}=3.31\frac{ML}{year.m}\#\left( 1 \right) \end{aligned}$

Supplementary Table S2 contains the summarized statistics for the Quinns Rocks area including the SIM wells.

Supplementary Table S2: Summary of hydraulic parameter for the Quinns Rocks area, from Kretschmer and Degens ^20^. Full table in supplementary materials.

| **Quinns Rocks** | **K (m/day)** | **Q (ML/year)** | **Q (ML/year/m)** |
| --- | --- | --- | --- |
| Mean | 128.22 | 3484.56 | 3.31 |
| Std. Error | 20.46 | 235.38 | 0.17 |
| Median | 79.00 | 3566.00 | 3.30 |
| Mode | 47.00 | 706.15 | 0.52 |
| Std. Dev. | 106.34 | 498647.53 | 0.27 |
| Kurtosis | 0.94 | 2.10 | 3.02 |
| Skewness | 1.18 | 0.07 | 1.39 |
| Range | 420.00 | 2642.00 | 1.74 |
| Minimum | 7.00 | 2189.00 | 2.74 |
| Maximum | 427.00 | 4831.00 | 4.47 |
| Count | 27.00 | 9.00 | 9.00 |

Supplementary Table S3 contains individual well data relevant to the Quinns Rocks area.

Supplementary Table S3: Hydraulic testing of the shallow aquifer from the Quinns Rocks region. The SIM wells are bolded.

| **Year** | **Test-Type** | **Geology** | **T** | **b** | **K** | **BoreID** |
| --- | --- | --- | --- | --- | --- | --- |
| 1994 | 8-hour constant rate | Tamala Limestone | 1400 | 30 | 47 | Private |
| 1996 | 7-hour multi rate | Tamala Limestone | 2000 | 30 | 67 | Private |
| 1993 | 10-hour constant rate | Tamala Limestone | 1100 | 22 | 50 | Private |
| 1993 | Multi rate, 8-hour constant rate | Tamala Limestone | 1200 | 30 | 40 | Private |
| 1996 | Multi rate, 10-hour constant rate | Tamala Limestone | 1200 | 26 | 46 | Private |
| 1994 | Mulit rate, 6-hour constant rate | Tamala Limestone | 7000 | 30 | 233 | Private |
| **1991** | **Tide time-lag & amplitude** | **Tamala Limestone** | **207** | **30** | **7** | **SIM 2/90** |
| **1991** | **Tide time-lag & amplitude** | **Tamala Limestone** | **2154** | **31** | **69** | **SIM 1/90** |
| **1991** | **Tide time-lag & amplitude** | **Tamala Limestone** | **5308** | **31** | **171** | **SIM 3/90** |
| **1991** | **Tide time-lag & amplitude** | **Tamala Limestone** | **7300** | **31** | **235** | **SIM 6/90** |
| **1991** | **Tide time-lag & amplitude** | **Tamala Limestone** | **2507** | **31** | **81** | **SIM 5/90** |
| 1991 | Tide time-lag & amplitude | Tamala Limestone | 9758 | 31 | 315 | SKP-GREEN |
| 1991 | Tide time-lag & amplitude | Tamala Limestone | 1463 | 31 | 47 | SKP-WHITE |
| 1991 | Multi-rate, 24-hour constant rate | Tamala Limestone | 12824 | 30 | 427 | QC |
| 1990 | Multi-rate, 12-hour constant rate | Tamala Limestone | 1390 | 30 | 46 | QW |
| 1986 | 8-hour duration | Tamala Limestone | 402.7 | 30 | 13 | 1/86 |
| 1994 | 8-hour duration | Tamala Limestone | 9186 | 30 | 306 | Q150 (QM10) |
| 1994 | 8-hour duration | Tamala Limestone | 5741 | 30 | 191 | Q140 (QA10) |
| 1994 | 8-hour duration | Tamala Limestone | 3674 | 30 | 122 | Q10 (QX10) |
| 1990 | 12-hour duration | Tamala Limestone | 6991 | 30 | 233 | Q170 (QW10) |
| 1996 | 24-hour duration | Tamala Limestone | 1832 | 30 | 61 | Q40 (QL10) |
| 1997 | 24-hour duration | Tamala Limestone | 1021 | 30 | 34 | Q200 (QZ20) |
| 1998 | 10-hour duration | Tamala Limestone | 2296 | 30 | 77 | Q190 (QZ10) |
| 1997 | 24-hour duration | Tamala Limestone | 2358 | 30 | 79 | Q60 (QE10) |
| 1998 | 10-hour duration | Tamala Limestone | 5103 | 30 | 170 | Q180 (QW20) |
| 1998 | 10-hour duration | Tamala Limestone | 4275 | 30 | 142 | Q160 (QN10) |
| 1998 | 12-hour duration | Tamala Limestone | 4593 | 30 | 153 | Q20 (QS10) |

Supplementary Table S4 contains estimations of groundwater recharge relevant to the Quinns Rocks area.

Supplementary Table S4: Collation of the recharge parameters in ^Kretschmer and Degens 20^

| **Appendix** | **Comment** | **K** | **T** | **Ocean Discharge** | **Area** | **Rainfall Recharge** | **Recharge to flow-cell** |
| --- | --- | --- | --- | --- | --- | --- | --- |
|  |  | **(m/day)** | **(m²/day)** | **(ML/year)** | **(km²)** | **(mm/year)** | **(ML/year.m)** |
| B1 | 1976 estimate plus 1906 - 2011 recharge (757 mm/year) | 10 | 330 | 1536 | 7.7 | 653.25 | 2.74 |
| B2 | 1906 - 2011 recharge (757 mm/year) | 10 | 330 | 1251 | 6.7 | 2536 | 3.51 |
| B3 | 1975 - 2011 recharge (695 mm/year) | 10 | 330 | 1251 | 6.7 | 2328 | 3.31 |
| B4 | 2002 - 2011 recharge (630 mm/year) | 10 | 330 | 1251 | 6.7 | 2111 | 3.11 |
| B5 | Future wet rainfall predictions, 747 mm/year | 10 | 330 | 1251 | 6.7 | 2502 | 3.48 |
| B6 | Future median rainfall predictions, 691 mm/year | 10 | 330 | 1251 | 6.7 | 2315 | 3.30 |
| B7 | Future dry rainfall predictions, 623 mm/year | 10 | 330 | 1251 | 6.7 | 2087 | 3.09 |
| B8a | Varied Spearwood Sand K, 1975 - 2011 recharge (695 mm/year) | 5 | 165 | 626 | 6.7 | 2328 | 2.74 |
| B8b | Varied Spearwood Sand K, 1975 - 2011 recharge (695 mm/year) | 20 | 660 | 2503 | 6.7 | 2328 | 4.47 |

# GPR Processing Flow

Ground penetrating radar (GPR) is used at a site located west of Perth, to reveal the variation in near-shore geological facies progressing inland from the ocean (see Section 3.1, Figure 6). Processing is completed using the software package ReflexW ^23^. The steps are listed in Supplementary Table S5.

Supplementary Table S5: Processing steps and parameters used for the GPR image in Figure 9

| Processing Step | Parameters |
| --- | --- |
| Subtract-mean (dewow) | 4 (ns) |
| Static correction | - |
| Subtracting average | 200 (ns) |
| Energy decay | 0.01 |
| Bandpass frequency filter | 200/400/900/1500 |
| Topography migration | 0.11 m/ns |
| Bandpass frequency filter | 200/400/900/1500 |
| Correct 3D topography | 0.11 m/ns |
| Time-depth conversion | 0.11 m/ns |

# Comparison of EOS 80 with TDS via Evaporation

The International Equation of State of Seawater 1980 (EOS-80) ^24^ approximates the concentration of seawater to practical salinity units (PSU) using measurements of electrical conductivity (EC). A near-linear relationship exists between EC and the major constituent of seawater, dissolved Sodium Chloride ^25,26^. For reference, the conductivity of standard seawater at 15 degrees centigrade is 4.2914 S/m ^27,28^. For a further example, Tyler, et al. ^29^ demonstrate the electrical conductivity of the global ocean (including seasonal conductivity variations).

Evaporative (180°C) testing from SIM 6 in 2005 produces 360 mg/L total dissolved solids (TDS). EC measurements of the water sample are 660 uS/cm (25°C). For comparison with Walton ^26^, this yields the linear conversion factor K = 0.545 for the fresh groundwater. The equivalent estimate of mg/L using the EOS-80 scale is 0.32118 PSU (~321.18 mg/L). No evaporative tests exist for higher salinity measurements at the Quinns Rocks reference site.

Supplementary Table S6: Comparison of electrical conductivity (EC) to total dissolved salts (TDS) in SIM 6 after evaporative testing and relationship to EOS-80 conversion.

| SIM 6 EC  (μS/cm) | SIM 6 TDS  (mg/) | Linear  conversion | EOS-80  (mg/L) |
| --- | --- | --- | --- |
| 660 | 360 | 0.545 | 321.18 |

# Estimation of groundwater throughflow

Supplementary Figure S1 shows the potential estimates of groundwater throughflow using various points of measurement, such as the measured water level, the equivalent freshwater head, and the inter-well gradients between certain wells.

Supplementary Figure S1: Set of charts showing the potential variation in estimated groundwater throughflow derived from hydraulic gradients in a highly heterogeneous shallow coastal aquifer. **A,** the groundwater throughflow derived from hydraulic gradient analysis assuming a 200 m/day homogeneous aquifer for the SIM wells in 1994, before seawater intrusion has occurred, and in 2014, after seawater intrusion has occurred and suggesting relatively low groundwater throughflow. **B**, the equivalent freshwater head levels and associated line-of-best-fit. **C**, the measured water levels and associated line-of-best-fit showing relatively high groundwater throughflow, and **D**, the lines-of-best-fit taken across selected wells, showing significant variations in the estimated groundwater throughflow. This figure highlights the implications of both a heterogeneous distribution of hydraulic conductivity, as well as the potential uncertainty associated with hydraulic gradient analysis in a coastal environment.

Supplementary Figure S2 shows the estimated groundwater throughflow across the SIM wells throughout the monitoring period. Estimates before 2000 are similar given that seawater intrusion is not overt. After 2000, seawater is present in the SIM 1 and SIM 3 wells and strongly affects the equivalent freshwater head gradients.

Supplementary Figure S2:Chart showing the estimated groundwater throughflow using the hydraulic gradients computed from equivalent freshwater head (EFH) and measured water level (MWL) in the SIM well series at Quinns Rocks between 1990 and 2019.

# Effects of Model Refinement on Toe Position

This appendix contains the change in head and mass associated with increasing mess refinement. The modelled used is for a homogeneous aquifer 30 m thick and hydraulic conductivity of 130 m/d over an impermeable substrate (the mean measurement from the Quinns Rocks reference site). Groundwater through flow is set to 3.4 ML/year. Supplementary Figure S3 shows the different meshes and positions of the toe. There is negligible different in the geometry of the saline water interface or position of the toe as provided below. There is approximately one order of magnitude difference between run times for ‘A’, compared to ‘C’, below.

Supplementary Figure S3: Set of images showing the effect of mesh discretizaiton on the position on a simulated seawater wedge in a high hydraulic conductivity aquifer under approximately 3 ML/year-m of groundwater throughflow.

# Random fields python script

# -*- coding: utf-8 -*-

"""

Script to generate the random field distribution shown in Costall (2020)

@author: costall.alex@gmail.com

"""

import numpy as np

from matplotlib import ticker, pyplot as plt

from gstools import SRF, Exponential, transform as tf

from gstools.random import MasterRNG

import datetime as dt

def get_feflow_nodes(fid):

from pandas import read_csv

data = read_csv(fid, delim_whitespace = True)

data = data[data['Y'] < 1]

x, y = data.X.values, data.Y.values

return x, y

def make_fields(vartype, var, scale = 1, **kwargs):

fields = {}

model = Exponential(dim=2,

var=1,

len_scale=50,

anis = 1./10.,

angles=np.deg2rad(1),

)

for n,a in enumerate(var):

srf = SRF(model, seed = a)

field = srf([x, y])

tf.normal_to_arcsin(srf)

tf.normal_to_lognormal(srf)

tf.zinnharvey(srf, conn="high")

field = 100*np.exp(field)

fields[a] = field

print('Field: {}\n\t Mean: {}\n\t Max: {}\n\t Min: {}'.format(a, np.mean(field), np.max(field), np.min(field)))

return fields

def plot_fields(fields):

fig,ax = plt.subplots(len(fields.keys()), sharex = True, sharey = True)

fig.set_size_inches(18,12)

for n,f in enumerate(fields.items()):

im = ax[n].tricontourf(x, y, f[1], cmap = 'jet', norm = None, levels = 1000)

ax[n].set_aspect('equal', adjustable='box')

ax[n].tick_params(which = 'both', direction = 'in')

ax[n].xaxis.set_minor_locator(ticker.AutoMinorLocator(2))

ax[n].minorticks_on()

ax[n].yaxis.set_minor_locator(ticker.AutoMinorLocator(2))

ax[n].set_title(str(f[0]))

ax[n].set_xlabel('Distance (m)')

ax[n].set_ylabel('Elevation (mASL)')

plt.colorbar(im, ax = ax[n], orientation = 'vertical')

fig.set_tight_layout('tight')

return fig, ax

def export_fields(fields):

for f in fields:

print(f)

z = np.zeros((len(x),))

xyz = np.stack((x,y,z,fields[f].T)).T

fOut = 'GSTools_RandomField_Seed' + str(f) + '_CON_' + dt.datetime.now().strftime('%Y-%m-%d')+'.dat'

np.savetxt(fOut, xyz, header = "X \t Y \t Z \t COND", comments = "")

return

#%%

rng = np.random.RandomState(MasterRNG(20190312)())

fid = r"\\Egpsrv08-02\egp\Home\Staff\264401K\FEFLOW_KDrive_Backup\generic_aquifer_nodes_for_randomfields.dat"

x, y = get_feflow_nodes(fid)

seeds_ = [20191112, 20191113, 20191114, 20191115, 20191116]

fields = make_fields(vartype = 'seeds', var = seeds_, scale = 40)

fig, ax = plot_fields(fields)

export_fields(fields)

#plt.hist(fields[seeds_[-1]], bins = 100)

# Random fields statistics

The statistical description of the randomly generated hydraulic conductivity fields are found below in Supplementary Table S7. The mean parameters are approximately equal to that described by field analysis of the Quinns Rocks reference site.

Supplementary Table S7: Statistical distribution of hydraulic conductivity parameters used for the random fields

|  | **20191112** | **20191113** | **20191114** | **20191115** | **20191116** | **Homogeneous** | |  |
| --- | --- | --- | --- | --- | --- | --- | --- | --- |
| mean | 148.67 | 168.07 | 137.45 | 148.33 | 158.54 | 150 |  |  |
| std | 158.17 | 200.37 | 142.65 | 139.18 | 134.85 | 0 |  |  |
| min | 4.37 | 3.99 | 4.98 | 8.24 | 6.26 | 150 |  |  |
| 25% | 54.13 | 52.27 | 48.65 | 61.23 | 70.35 | 150 |  |  |
| 50% | 103.35 | 102.97 | 91.10 | 108.63 | 120.75 | 150 |  |  |
| 75% | 182.60 | 204.36 | 171.68 | 186.81 | 202.87 | 150 |  |  |
| max | 2078.62 | 2093.58 | 1689.60 | 2699.03 | 2204.99 | 150 |  |  |

# Inversion Parameters for RES2DINV

Supplementary Table S8 contains the inversion parameters used for inversion of synthetic and field data throughout the manuscript.

Supplementary Table S8: Inversion parameters used for RES2DINV (version 4.09).

| **Parameter** | **Value** |
| --- | --- |
| Initial damping factor (0.01 to 1.00) | 0.15 |
| Minimum damping factor (0.001 to 0.75) | 0.02 |
| Local optimization option (0=No, 1=Yes) | 1 |
| Convergence limit for relative change in RMS error in percent (0.1 to 20) | 5 |
| Minimum change in RMS error for line search in percent (0.5 to 100) | 0.5 |
| Number of iterations (1 to 30) | 7 |
| Vertical to horizontal flatness filter ratio (0.25 to 4.0) | 1 |
| Model for increase in thickness of layers (0=default 10%, 1=default 25%, 2=user defined) | 2 |
| Number of nodes between adjacent electrodes (1, 2 or 4) | 4 |
| Flatness filter type, Include smoothing of model resistivity (0=model changes only,1=directly on model) | 1 |
| Reduce number of topographical data points? (0=No,1=Yes. Recommend leave at 0) | 0 |
| Carry out topography modelling? (0=No,1=Yes) | 1 |
| Type of topography trend removal (0=Average,1=Least-squares,2=End to end) | 0 |
| Type of Jacobian matrix calculation (0=Quasi-Newton, 1=Gauss-Newton, 2=Mixed) | 1 |
| Increase of damping factor with depth (1.0 to 2.0) | 1.05 |
| Type of topographical modelling (0=None, 1=No longer supported so do not use, 2=uniform distorted FEM, 3=underwater, 4=damped FEM, 5=FEM with inverse Swartz-Christoffel) | 4 |
| Robust data constrain? (0=No, 1=Yes) | 1 |
| Cut-off factor for data constrain (0.0001 to 0.1)) | 0.05 |
| Robust model constraint? (0=No, 1=Yes) | 1 |
| Cut-off factor for model constrain (0.0001 to 1.0) | 0.005 |
| Allow number of model parameters to exceed data points? (0=No, 1=Yes) | 1 |
| Use extended model? (0=No, 1=Yes) | 0 |
| Reduce effect of side blocks? (0=No, 1=Slight, 2=Severe, 3=Very Severe) | 2 |
| Type of mesh (0=Normal,1=Fine,2=Finest) | 2 |
| Optimise damping factor? (0=No, 1=Yes) | 0 |
| Time-lapse inversion constrain (0=None,1&2=Smooth,3=Robust) | 3 |
| Type of time-lapse inversion method (0=Simultaneous,1=Sequential) | 0 |
| Thickness of first layer (0.25 to 1.0) | 0.25 |
| Factor to increase thickness layer with depth (1.0 to 1.25) | 1 |
| Use finite element method (yes=1,no=0) | 1 |
| Width of blocks (1=normal width, 2=double, 3=triple, 4=quadruple, 5=quintuple) | 1 |
| Make sure blocks have the same width (yes=1,no=0) | 0 |
| Rms convergence limit (in percent) | 0.1 |
| Use logarithm of apparent resistivity (0=use log of apparent resistivity, 1=use resistance values, 2=use apparent resistivity) | 0 |
| Type of ip inversion method (0=concurrent,1=sequential) | 0 |
| Proceed automatically for sequential method (1=yes,0=no) | 0 |
| IP DAMPING FACTOR (0.01 to 1.0) | 0.25 |
| Use automatic ip damping factor (yes=1,no=0) | 0 |
| CUTOFF FACTOR FOR BOREHOLE DATA (0.0005 to 0.02) | 0.0001 |
| TYPE OF CROSS-BOREHOLE MODEL (0=normal,1=half size) | 0 |
| Limit resistivity values(0=no,1=yes) | 1 |
| Upper limit factor (10-50) | 50 |
| Lower limit factor (0.02 to 0.1) | 0.02 |
| Type of reference resistivity (0=average,1=first iteration) | 1 |
| Model refinement (1.0=Normal,0.5=Half-width cells) | 0.5 |
| Combined Marquardt and Occam inversion (0=Not used,1=used) | 1 |
| Type of optimisation method (0=Gauss-Newton,2=Incomplete GN) | 0 |
| Convergence limit for Incomplete Gauss-Newton method (0.005 to 0.05) | 0.005 |
| Use data compression with Incomplete Gauss-Newton (0=No,1=Yes) | 0 |
| Use reference model in inversion (0=No,1=Yes) | 1 |
| Damping factor for reference model (0.0 to 1.0) | 0.01 |
| Use fast method to calculate Jacobian matrix. (0=No,1=Yes) | 0 |
| Use higher damping for first layer? (0=No,1=Yes) | 1 |
| Extra damping factor for first layer (1.0 to 100.0) | 5 |
| Type of finite-element method (0=Triangular,1=Trapezoidal elements) | 1 |
| Factor to increase model depth range (1.0 to 5.0) | 1.05 |
| Reduce model variations near borehole (0=No, 1=Yes) | 0 |
| Factor to control the degree variations near the boreholes are reduced (2 to 100) | 5 |
| Factor to control variation of borehole damping factor with distance (0.5 to 5.0) | 1 |
| Floating electrodes survey inversion method (0=use fixed water layer, 1=Incorporate water layer into the model) | 1 |
| Resistivity variation within water layer (0=allow resistivity to vary freely,1=minimise variation) | 1 |
| Use sparse inversion method for very long survey lines (0=No, 1=Yes) | 0 |
| Optimize Jacobian matrix calculation (0=No, 1=Yes) | 0 |
| Automatically switch electrodes for negative geometric factor (0=No, 1=Yes) | 1 |
| Force resistance value to be consistent with the geometric factor (0=No, 1=Yes) | 0 |
| Shift the electrodes to round up positions of electrodes (0=No, 1=Yes) | 0 |
| Use difference of measurements in time-lapse inversion (0=No,1=Yes) | 0 |
| Use active constraint balancing (0=No,1=Yes) | 0 |
| Type of active constraints (0=Normal,1=Reverse) | 0 |
| Lower damping factor limit for active constraints | 0.4 |
| Upper damping factor limit for active constraints | 2.5 |
| Water resistivity variation damping factor | 8 |
| Use automatic calculation for change of damping factor with depth (0=No,1=Yes) | 0 |
| Type of I.P. model transformation (0=None, 1=square root, 3=range) | 1 |
| Model Chargeability Lower Limit (mv/V) for range | 0 |
| Model Chargeability Upper Limit (mv/V) for range | 900 |
| Use I.P. model refinement (0=No, 1=Yes) | 1 |
| Weight for I.P. data (1 to 10) | 1 |
| I.P. model damping factor (0.05 to 1.0) | 0.25 |
| Use program estimate for I.P. model damping factor (0=No, 1=Yes) | 0 |
| Type of I.P. smoothness constraint (1=Same as resistivity, 0=Different) | 1 |
| Joint or separate I.P. inversion method (1=Separate, 0=Joint) | 1 |
| Apparent I.P. cut off value (300 to 899 mv/V) | 899 |
| Use diagonal filter (0=No, 1=Yes) | 1 |
| Diagonal filter weight (0.2 to 5.0) | 1 |
| Limit range of data weights from error estimates? (0=No, 1=Yes) | 0 |
| Lower limit of data weights (0.2 to 0.5) | 0.3 |
| Upper limit of data weights (2.0 to 5.0) | 3 |
| Use same data weights from error estimates for different time series? (0=No, 1=Yes) | 0 |
| Calculate model resolution? (0=No, 1=Yes) | 1 |
| Use L curve method? (0=No, 1=Yes) | 0 |
| Use same norms in L curve method? (0=No, 1=Yes) | 0 |
| Allow damping factor in increase in L curve method? (0=No, 1=Yes) | 1 |
| Type of borehole damping method (0=Horizontal distance from nearest borehole, 1=Distance from nearest active electrode) | 0 |
| Use fast Jacobian calculation for dense data sets? (0=No,1=Yes) | 0 |
| Use higher damping factors at sides of model? (0=No,1=Yes) | 1 |
| Adjust damping factors for distances between the blocks in the model? (0=No,1=Yes) | 1 |
| Number of electrodes in segment for sparse inversion method for very long survey lines. | 250 |
| Time-lapse damping factor. | 0.25 |
| Reduce time-lapse damping with each iteration? (0=No,1=Yes) | 1 |
| Filter input data using geometric factor? (0=No,1=Yes) | 0 |
| Automatically remove negative apparent resistivity values? (0=No,1=Yes) | 0 |
| Automatically remove Gamma type arrays? (0=No,1=Yes) | 0 |

# References Cited within Supplementary

1 Abd-Elhamid, H. & Javadi, A. An investigation into control of saltwater intrusion considering the effects of climate change and sea level rise. *Proceeding of 20th SWIM* **2327** (2008).

2 Chang, S. W., Clement, T. P., Simpson, M. J. & Lee, K.-K. Does sea-level rise have an impact on saltwater intrusion? *Advances in Water Resources* **34**, 1283-1291, doi:10.1016/j.advwatres.2011.06.006 (2011).

3 Ketabchi, H., Mahmoodzadeh, D., Ataie-Ashtiani, B. & Simmons, C. T. Sea-level rise impacts on seawater intrusion in coastal aquifers: Review and integration. *Journal of Hydrology* **535**, 235-255, doi:10.1016/j.jhydrol.2016.01.083 (2016).

4 Masterson, J. P. *Simulated interaction between freshwater and saltwater and effects of ground-water pumping and sea-level change, Lower Cape Cod aquifer system, Massachusetts*. (US Department of the Interior, US Geological Survey, 2004).

5 Werner, A. D. A review of seawater intrusion and its management in Australia. *Hydrogeology Journal* **18**, 281-285, doi:10.1007/s10040-009-0465-8 (2010).

6 Werner, A. D. & Simmons, C. T. Impact of sea-level rise on sea water intrusion in coastal aquifers. *Ground Water* **47**, 197-204, doi:10.1111/j.1745-6584.2008.00535.x (2009).

7 Ataie-Ashtiani, B., Volker, R. E. & Lockington, D. A. Tidal effects on sea water intrusion in unconfined aquifers. *Journal of Hydrology* **216**, 17-31, doi:Doi 10.1016/S0022-1694(98)00275-3 (1999).

8 Pool, M., Post, V. E. A. & Simmons, C. T. Effects of tidal fluctuations on mixing and spreading in coastal aquifers: Homogeneous case. *Water Resources Research* **50**, 6910-6926, doi:10.1002/2014wr015534 (2014).

9 Heiss, J. W. & Michael, H. A. Saltwater-freshwater mixing dynamics in a sandy beach aquifer over tidal, spring-neap, and seasonal cycles. **50**, 6747-6766, doi:10.1002/2014wr015574 (2014).

10 Abarca, E., Karam, H., Hemond, H. F. & Harvey, C. F. Transient groundwater dynamics in a coastal aquifer: The effects of tides, the lunar cycle, and the beach profile. *Water Resources Research* **49**, 2473-2488, doi:10.1002/wrcr.20075 (2013).

11 Sebben, M. L., Werner, A. D. & Graf, T. Seawater intrusion in fractured coastal aquifers: A preliminary numerical investigation using a fractured Henry problem. *Advances in Water Resources* **85**, 93-108, doi:10.1016/j.advwatres.2015.09.013 (2015).

12 Wicks, C. & Herman, J. The Effect of Zones of High Porosity and Permeability on the Configuration of the Saline‐Freshwater Mixing Zone. *Groundwater* **33**, 733-740, doi:doi:10.1111/j.1745-6584.1995.tb00019.x (1995).

13 Gelhar, L. W., Welty, C. & Rehfeldt, K. R. A critical review of data on field-scale dispersion in aquifers. **28**, 1955-1974, doi:doi:10.1029/92WR00607 (1992).

14 Schulze-Makuch, D. Longitudinal dispersivity data and implications for scaling behavior. *Ground Water* **43**, 443-456, doi:10.1111/j.1745-6584.2005.0051.x (2005).

15 Shoemaker, W. B. Important observations and parameters for a salt water intrusion model. *Ground Water* **42**, 829-840, doi:10.1111/j.1745-6584.2004.t01-2-.x (2004).

16 Volker, R. E. & Rushton, K. R. An assessment of the importance of some parameters for seawater intrusion in aquifers and a comparison of dispersive and sharp-interface modelling approaches. *Journal of Hydrology* **56**, 239-250, doi:<http://dx.doi.org/10.1016/0022-1694(82)90015-4> (1982).

17 Walther, M., Graf, T., Kolditz, O., Liedl, R. & Post, V. How significant is the slope of the sea-side boundary for modelling seawater intrusion in coastal aquifers? *Journal of Hydrology* **551**, 648-659, doi:<https://doi.org/10.1016/j.jhydrol.2017.02.031> (2017).

18 Hussain, M. S. & Javadi, A. A. Assessing impacts of sea level rise on seawater intrusion in a coastal aquifer with sloped shoreline boundary. *Journal of Hydro-Environment Research* **11**, 29-41, doi:10.1016/j.jher.2016.01.003 (2016).

19 Abarca, E., Carrera, J., Sánchez-Vila, X. & Voss, C. I. Quasi-horizontal circulation cells in 3D seawater intrusion. *Journal of Hydrology* **339**, 118-129, doi:<https://doi.org/10.1016/j.jhydrol.2007.02.017> (2007).

20 Kretschmer, P. & Degens, B. Vol. Hydrogeological Report Series (ed Western Australian Department of Water) (2012).

21 Nidagal, V. & Davidson, W. J. G. S. o. W., Unpublished Hydrogeological Report. North coastal groundwater investigation (Burns Beach—Pipidinny). (1991).

22 Diersch, H.-J. G. *Feflow: Finite Element Modeling of Flow, Mass and Heat Transport in Porous and Fractured Media*. (Springer Science & Business Media, 2014).

23 Sandmeir, K. J. ReflexW (Manual). (2014).

24 UNESCO, I. J. U. T. P. M. S. Tenth report of the joint panel on oceanographic tables and standards. **36**, 15-19 (1981).

25 Arps, J. The effect of temperature on the density and electrical resistivity of sodium chloride solutions. *Journal of Petroleum Technology* **5**, 17-20 (1953).

26 Walton, N. R. G. Electrical Conductivity And Total Dissolved Solids—What Is Their Precise Relationship? *Desalination* **72**, 275-292 (1989).

27 Culkin, F. & Smith, N. J. I. J. o. O. E. Determination of the concentration of potassium chloride solution having the same electrical conductivity, at 15 C and infinite frequency, as standard seawater of salinity 35.0000‰(Chlorinity 19.37394‰). **5**, 22-23 (1980).

28 Millero, F. J., Feistel, R., Wright, D. G. & McDougall, T. J. The composition of Standard Seawater and the definition of the Reference-Composition Salinity Scale. *Deep-Sea Research Part I-Oceanographic Research Papers* **55**, 50-72, doi:10.1016/j.dsr.2007.10.001 (2008).

29 Tyler, R. H. *et al.* Electrical conductivity of the global ocean. **69**, 156 (2017).
